# Transferrin receptor 1 nuclear translocation facilitates tumor progression via p53-mediated chromatin interactions and genome-wide alterations

**DOI:** 10.1038/s41392-025-02297-6

**Published:** 2025-07-08

**Authors:** Yaxin Hou, Guoheng Tang, Qizhi Wang, Meng Zhou, Ran Xu, Xuehui Chen, Guizhi Shi, Zhuoran Wang, Xiyun Yan, Jie Zhuang, Kelong Fan

**Affiliations:** 1 CAS Engineering Laboratory for Nanozyme, Key Laboratory of Biomacromolecules, CAS Center for Excellence in Biomacromolecules, Institute of Biophysics, Chinese Academy of Sciences, Beijing, China; 2 University of Chinese Academy of Sciences, Chinese Academy of Sciences, Beijing, China; 3 State Key Laboratory of Stem Cell and Reproductive Biology, Institute of Stem Cell and Regeneration, Beijing Institute of Stem Cell and Regenerative Medicine, Institute of Zoology, Chinese Academy of Sciences, Beijing, China; 4 Laboratory Animal Center of Institute of Biophysics, Chinese Academy of Sciences, Aviation General Hospital of Beijing, University of Chinese Academy of Sciences, Beijing, China; 5 Nanozyme Laboratory in Zhongyuan, Henan Academy of Innovations in Medical Science, Zhengzhou, China; 6 School of Medicine, Nankai University, Tianjin, China

**Keywords:** Oncogenes, Cell biology

## Abstract

Transferrin receptor 1 (TfR1), a widely expressed type II transmembrane glycoprotein located on the plasma membrane, is well known for its established role in cellular iron uptake. Nevertheless, emerging evidence implies that TfR1 exhibits previously unrecognized noncanonical functions. Herein, we demonstrated the nuclear translocation of TfR1 and revealed the interaction between TfR1 and p53 within the nucleus. Through comprehensive analyses at the proteomic, genomic, and transcriptomic levels, we demonstrated that this interaction significantly influences the transcriptional activity of p53 on its downstream target genes, which are highly enriched in DNA damage repair functions. Specifically, our investigation revealed the indispensable role of nuclear TfR1 in the regulation of the nucleotide excision repair (NER) pathway, exemplified by the transcriptional regulation of *XPC*. Notably, both in vitro and in vivo results revealed a positive regulatory role of TfR1 in the NER pathway. Subsequent phenomic analysis of clinical colorectal tumor samples confirmed a positive correlation between nuclear TfR1 levels and tumor malignancy, aggressive features, and metastasis. Collectively, our findings highlight the non-classical function of TfR1, emphasizing its importance in the regulation of gene expression, as well as tumor progression.

## Introduction

As vital constituents of the cellular membrane, plasma membrane proteins play crucial roles in substance transportation, signal transduction, ion exchange, and energy conversion and have long been a focal point of research in the life sciences.^1^ Transferrin receptor 1 (TfR1), also known as CD71, is a dimeric type II transmembrane glycoprotein that is widely expressed across various species.^2^ Classically, TfR1 mediates iron uptake by binding iron-loaded transferrin. Therefore, TfR1 is involved in diverse biological processes due to the vital importance of iron in cellular activities.^3^ For example, TfR1-mediated iron metabolism plays a central role in erythropoiesis regulation.^4^ Moreover, TfR1 is overexpressed in various types of tumors, where it contributes to tumor progression and has been identified as an important target for tumor diagnosis and treatment.^5–9^ According to the Gene Expression Profiling Interactive Analysis (GEPIA) database, colorectal cancer (CRC) exhibits one of the most pronounced increases in TfR1 expression relative to normal tissues.^10^ Notably, CRC is currently the third most commonly diagnosed cancer in the world, and based on theoretical projections, its global incidence is expected to rise dramatically, potentially ranking first by 2070.^11,12^ In this context, growing research efforts have focused on elucidating the role of TfR1 in CRC progression. For example, TfR1-mediated iron uptake has been demonstrated to facilitate CRC cell proliferation through activation of the β-catenin/c-Myc/E2F1/POLD1 signaling pathway.^13^ Although the classical function of TfR1 has been comprehensively studied, ongoing research on its non-classical functions persists, as exemplified by the activation of the NF-κB signaling pathway via interacting with the IκB kinase complex,^14^ the regulation of gene expression through alternative splicing,^15–17^ the maintenance of intestinal homeostasis,^18^ and so forth. These findings indicate that TfR1 has diverse, unexplored functions. However, the molecular mechanisms by which TfR1 performs non-classical functions are still poorly understood.

Numerous plasma membrane proteins have been elucidated to localize in the nucleus. Wherein, the majority of these plasma membrane proteins belong to receptors.^19–21^ Several transport pathways have been identified that mediate the translocation of membrane proteins from the plasma membrane to the nucleus. One canonical pathway involves retrograde trafficking from the Golgi complex to the endoplasmic reticulum (ER), followed by translocation into the nucleus, exemplified by various members of the receptor tyrosine kinase (RTK) family, such as fibroblast growth factor receptor 1.^22^ Another notable pathway relies on the microtubule-dependent trafficking of vesicles to the nucleus via sorting nexins, a mechanism that has been demonstrated in several G-protein coupled receptor proteins.^23^ Once translocated to the nucleus, these plasma membrane receptors frequently acquire novel functions distinct from their canonical identities via interaction with other nuclear proteins. For instance, the insulin receptor has been demonstrated to act as a transcriptional cofactor to regulate downstream genes via the association with RNA polymerase II and coregulator host cell factor-1 after nuclear translocation, leading to the activation of insulin-induced cell proliferation and triglyceride accumulation.^24^ In addition, the epidermal growth factor receptor was reported to bind with transcriptional intermediary factor 2 in the nucleus, transcriptionally regulating cyclin D1 expression and thereby promoting the cell-cycle progression and proliferation of tumor cells.^25^ These discoveries collectively highlight that alterations in the subcellular localization of proteins directly affect their function and underscore the importance of the nuclear translocation of plasma membrane proteins, opening a new research avenue.

p53 is a pivotal transcription factor that specifically binds to DNA and transcriptionally regulates a variety of genes, participating in the regulation of various signaling pathways, including DNA damage repair, cell-cycle progression, senescence, and apoptosis.^26^ p53 plays a crucial role in promoting tumor progression through a series of mechanisms.^27–29^ Among these mechanisms, the DNA damage repair pathway plays a crucial role since it is critical for tumor cells to withstand lethal lesions to DNA.^30–32^ For example, p53-mediated transcription of DNA damage repair-related genes has been validated to promote the survival of prostate carcinoma cells after exposure to ionizing radiation.^33^ Moreover, the activation of p53 downstream genes associated with the nucleotide excision repair (NER) pathway, including *XPC*, *XPA*, *ERCC1*, *ERCC2*, *ERCC5*, and *DDB2*, leads to increased resistance to interstrand cross-link damage and facilitates survival in glioma cells.^34^ These studies indicate that p53 is a critical target for investigating the molecular mechanisms of tumors.

In this study, we demonstrated the nuclear localization of TfR1 in vitro and in vivo and explored its nuclear translocation mechanism. Proteomic data revealed the interaction between TfR1 and p53, which was further confirmed through co-immunoprecipitation (Co-IP) and surface plasmon resonance. Genomic analyses revealed that nuclear TfR1 at gene promoters is associated with p53. Transcriptomic analysis confirmed that TfR1 regulates the transcription of DNA damage repair-related genes through the interaction of p53. More importantly, the phenomic results from a clinical tumor tissue microarray correlated elevated nuclear TfR1 with tumor malignancy, aggressiveness, and metastasis in CRC. These findings provide novel insights into the functions of TfR1 in promoting tumor progression, particularly through its nuclear translocation.

## Results

### TfR1 localizes to cell nucleus

**Fig. 1:**
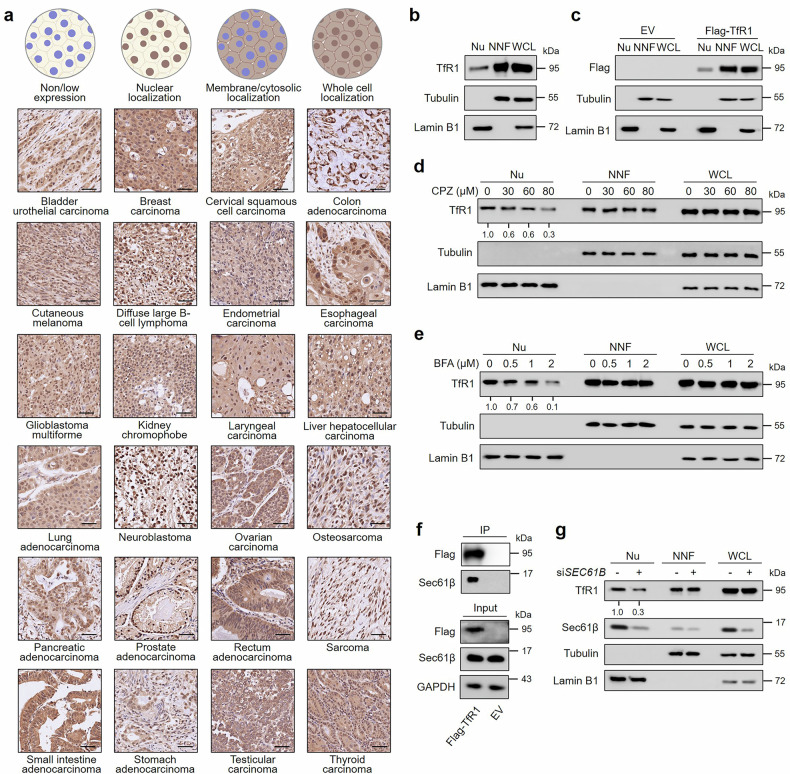
Identification of the nuclear localization of TfR1 and characterization of its nuclear translocation mechanism. **a** Representative IHC images (×10 magnification) of TfR1 staining in tumor tissues from different types of cancer (*n* = 204). Scale bar = 50 μm. **b**, **c** Fractionation of the nuclear and cytoplasmic components of HCT-116 cells in (**b**) and HCT-116 cells transfected with empty vector (EV) or Flag-TfR1 in (**c**). Lamin B1 was used as a nuclear (Nu) loading control. Tubulin was used as non-nuclear fraction (NNF), and whole cell lysate (WCL) loading control. **d**, **e** HCT-116 cells were treated with different concentrations of CPZ for 30 min in (**d**) or BFA for 6 h in (**e**) for immunoblot analysis of nuclear and cytoplasmic fractions. The relative abundance of nuclear TfR1 was normalized to that of the CPZ or BFA = 0 controls. Lamin B1 was used as a Nu loading control. Tubulin was used as the loading control for NNF and WCL. **f** Co-IP analysis of the interactions between Flag-TfR1 and Sec61β. GAPDH was used as a loading control. **g** HCT-116 cells were transfected with scrambled negative control siRNA (-) or *SEC61B*-targeted siRNA (+) for 72 h, followed by subcellular fractionation and immunoblot analysis. The relative abundance of nuclear TfR1 was normalized to that of the siRNA (-) control. Lamin B1 and Tubulin were used as equal loading controls for Nu, NNF, and WCL

Previously, our group identified the nuclear localization of TfR1 in tumor tissues by employing magnetoferrin, which consists of iron oxide nanoparticles and heavy-chain ferritin (a ligand of TfR1), for histological staining.^35^ To validate this phenomenon more comprehensively, we developed a panel of monoclonal antibodies against the intracellular domain of human TfR1 via hybridoma technology, the specificity of which was confirmed through Co-IP assay and flow cytometry. As shown in Supplementary Fig. 1, 23B10 specifically bound to human TfR1. To confirm the nuclear localization of TfR1, we performed immunohistochemistry (IHC) on a multi-organ cancer tissue microarray and confirmed that TfR1 was localized in the nucleus of 24 types of human tumors (Fig. 1a), with up to an 88.7% positive rate of nuclear staining (Supplementary Fig. 2). Moreover, we explored the subcellular localization of TfR1 in vitro via numerous approaches. Immunofluorescence confocal microscopy clearly demonstrated the nuclear localization of TfR1 in various tumor cell lines, including HCT-116 (human colorectal carcinoma), A549 (human lung carcinoma), Hep G2 (human hepatic carcinoma), and U-87 MG (human glioma) (Supplementary Fig. 3). Additionally, to ascertain the nuclear localization of TfR1 was not due to nonspecific binding of the anti-TfR1 antibody, we transfected HCT-116 cells with EGFP-fused TfR1. Clearly, fluorescence signals were observed in the cell nucleus via confocal microscopy (Supplementary Fig. 4). Moreover, by fractionation of nuclear and cytoplasmic components, both endogenously expressed TfR1 and exogenously expressed Flag-TfR1 in the nucleus were detected (Fig. 1b, c). To further validate the accuracy of this result, we examined markers of the ER (Calreticulin) and Golgi complex components (Syntaxin-6) in the nuclear fraction. As shown in Supplementary Fig. 5, TfR1 was detected in the nucleus, whereas markers of these membrane components were absent, indicating that there was no contamination.

Given the observed nuclear localization of TfR1 in clinical tumor specimens and in tumor cell lines, we investigated the molecular mechanism through which TfR1 is translocated from the cellular membrane to the nucleus. First, we hypothesized that blockage of the common subcellular trafficking pathway of TfR1 might prevent its nuclear translocation. Since TfR1 is internalized primarily via clathrin-mediated endocytosis, we utilized chlorpromazine (CPZ), a well-known inhibitor of this process.^36^ Evidently, CPZ efficiently attenuated the fraction of TfR1 in the nucleus in a concentration-dependent manner (Fig. 1d, Supplementary Fig. 6). Next, we investigated how TfR1 trafficked to the nucleus after being internalized from the membrane. One of the important routes of nuclear translocation for plasma membrane receptors is the Golgi complex–ER retrotranslocation through coatomer protein I-coated vesicles after internalization.^37^ Additionally, approximately 10% of the internalized TfR1 is recycled through the Golgi complex.^38^ Hence, we speculated that TfR1 has the potential to be trafficked to the nucleus through the Golgi complex–ER retrotranslocation pathway. To verify this hypothesis, brefeldin A (BFA) was used for the disassembly of coatomer protein I-coated vesicles.^39^ With increasing concentrations of BFA, the fraction of nuclear TfR1 tended to decrease (Fig. 1e, Supplementary Fig. 7). Thus, we demonstrated that TfR1 can be translocated to the nucleus via a retrograde route from the Golgi complex to the ER. Sec61β, an important subunit of the ER translocon, has been reported to mediate the retrotranslocation of various plasma membrane proteins from the ER to the nucleus.^40–42^ Therefore, we assessed the interaction between TfR1 and Sec61β via Co-IP. As shown in Fig. 1f, TfR1 was shown to interact with Sec61β. Subsequently, we aimed to explore the involvement of Sec61β in the TfR1 translocation process. As expected, knockdown of Sec61β by siRNA significantly inhibited the nuclear translocation of TfR1 (Fig. 1g, Supplementary Fig. 8). Thus far, we have demonstrated a retrograde route of nucleus-localized TfR1 from the plasma membrane to the nucleus. Next, we investigated the mechanism by which TfR1 is imported through the nuclear pore complex. To this end, we validated the interaction of TfR1 with several classical nuclear transporters (importin-α, importin-β1, and transportin-1).^43,44^ Co-IP assays revealed that TfR1 can bind to all these nuclear transport machineries (Supplementary Fig. 9).

We subsequently confirmed that mutating a putative nuclear localization signal (KPKR) within the intracellular domain (ICD)^45^ or deleting the entire ICD of TfR1 did not impede the nuclear translocation of TfR1 (Supplementary Fig. 10). These results indicated that the ICD of TfR1 may not be critical for its nuclear transport. Taken together, these findings support the nuclear localization of TfR1 and suggest potential mechanisms mediating its translocation to the nucleus.

### TfR1 interacts with p53 in the nucleus

**Fig. 2:**
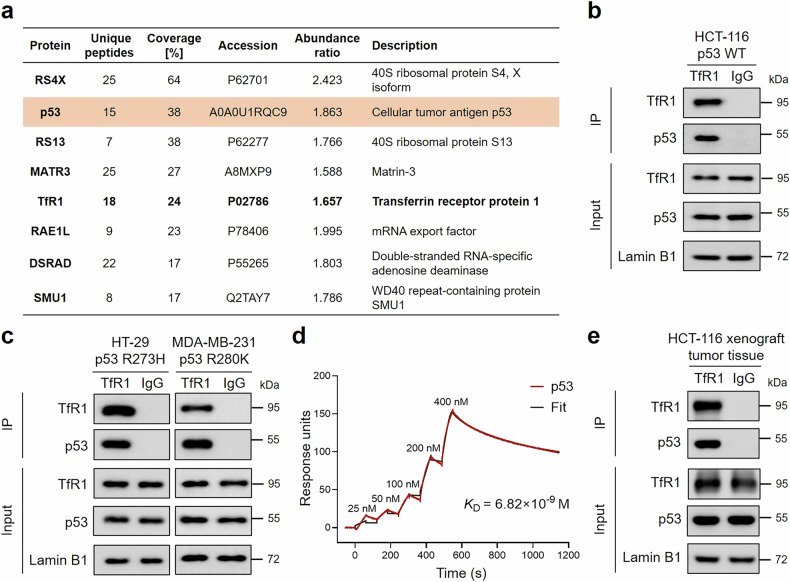
Proteomic data reveal the interaction between TfR1 and p53 in the nucleus. **a** The top-ranking proteins identified by mass spectrometry. p53 was highlighted in orange. **b**, **c** Co-IP analysis of the interaction between TfR1 and p53 in the nuclei of HCT-116 cells (**b**), HT-29 cells, and MDA-MB-231 cells (**c**). **d** Measurement of TfR1 binding to p53 with single-cycle kinetics using BIAcore. **e** Co-IP was used to detect the association of TfR1 with p53 in the nucleus of HCT-116 xenograft tumor tissues. Lamin B1 was used as a loading control

Owing to the lack of a DNA-binding motif in TfR1,^46,47^ the mechanism by which TfR1 executes its biological function in the nucleus is unclear. An unbiased approach to capture the potential associations in the nucleus was designed to further investigate the interaction of cell membrane receptors with nuclear proteins. We performed immunoprecipitation and mass spectrometry to screen for potential TfR1-associated proteins (Fig. 2a). To validate the mass spectrometric results, we sequentially carried out Co-IP assays of the proteins scoring from high to low. The interaction between TfR1 and RS4X (the top-ranked protein) was not detected, but we fortunately identified the interaction between TfR1 and p53, which ranked second in the list (Fig. 2b and Supplementary Fig. 11). Furthermore, p53 is frequently mutated in cancer cells, leading to functional alterations.^48^ Consequently, in addition to conducting Co-IP in a p53 wild-type cell line (HCT-116), we further investigated other p53 mutant cell lines, including HT-29 (human colorectal carcinoma) and MDA-MB-231 (human breast carcinoma) cells. Intriguingly, we observed the interaction in these cell lines harboring different p53 mutations, suggesting relative conservation of the binding sites of TfR1 and p53 (Fig. 2c). Furthermore, using the biomolecular interaction analysis core-technology (BIAcore) surface plasmon resonance assay with recombinant purified TfR1 and p53 proteins (Supplementary Fig. 12), we demonstrated that the interaction between TfR1 and p53 was direct and strong (*K*_D_ = 6.82 × 10^−^^9 ^M) (Fig. 2d). To simulate these conditions in vivo, we established an HCT-116 tumor-bearing nude mouse model in which Co-IP further demonstrated the interaction between TfR1 and p53 in tumor tissues (Fig. 2e). To further investigate the molecular mechanism underlying the interaction between p53 and TfR1, Co-IP analysis was performed using p53 truncations or point mutants. As shown in Supplementary Fig. 13, the binding region was mapped to amino acids 51–53 of p53. Overall, we speculated that p53 might be a crucial mediator for TfR1 functioning in the nucleus.

### Nuclear TfR1 binds to gene promoters associated with p53

**Fig. 3:**
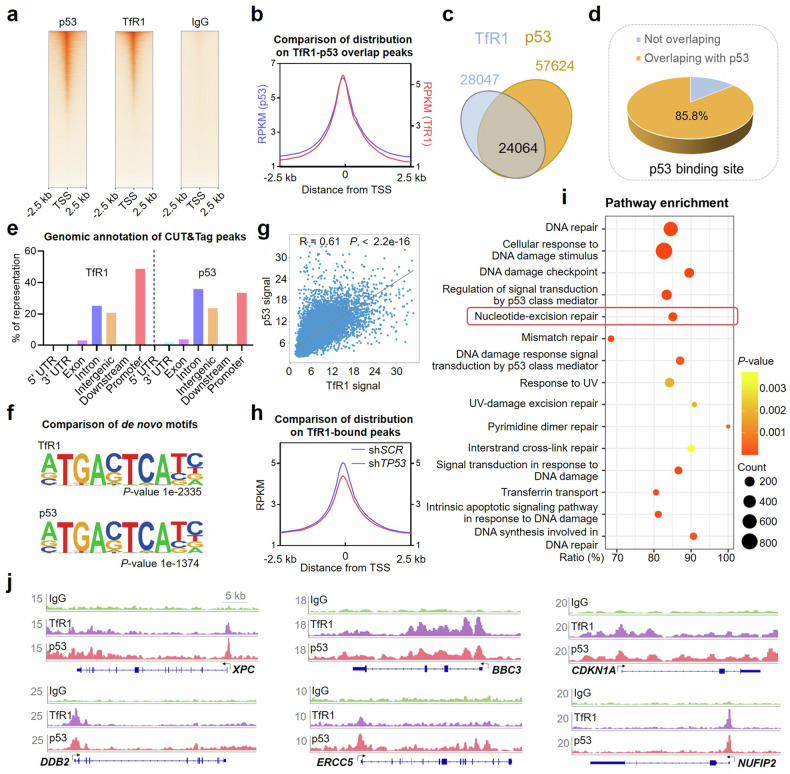
Genome-wide analysis revealed that nuclear TfR1-binding gene promoters are strongly associated with p53. **a** Heatmaps of p53 and TfR1 CUT&Tag peaks near the TSSs in HCT-116 cells obtained via anti-p53 and anti-TfR1 antibodies, respectively. IgG served as a control. Raw read densities were used. Each horizontal line indicates an individual gene locus. **b** CUT&Tag read density plot for p53 and TfR1 at TfR1-bound gene loci. **c** Venn diagram overlap analysis of p53 and TfR1 peaks. **d** Pie chart analysis of the proportion of TfR1 peaks overlapping with p53. **e** TfR1 and p53 peaks classified by human genomic annotations (hg38). **f** Top consensus motifs identified by de novo motif discovery at the TfR1 and p53 sites on promoters. **g** Linear correlation between the signals of the p53 and TfR1 peaks. R, the coefficient of determination of the two correlations. **h** CUT&Tag read density plot for sh*SCR* and sh*TP53* cells at TfR1-bound gene loci. **i** Dot plot of the GO term enrichment analysis results for the TfR1 peaks. **j** CUT&Tag peak distribution for TfR1 at representative gene loci

With strong evidence of the interaction between TfR1 and p53 in the nucleus, we subsequently compared the genome-wide binding patterns of TfR1 and p53 using cleavage under targets and tagmentation (CUT&Tag) assays with anti-TfR1 and anti-p53 antibodies, respectively. Strikingly, CUT&Tag for TfR1 identified 28,047 high-confidence peaks in HCT-116 cells, which were prominently enriched near the transcription start sites (TSSs) (Fig. 3a, b). However, knockdown of TfR1 resulted in a significant reduction in the intensity of the TfR1 peaks (Supplementary Fig. 14). Remarkably, up to 85.8% of the TfR1 peaks overlapped with the p53 peaks (Fig. 3c, d), and the peak distributions of p53 and TfR1 exhibited a high degree of resemblance in the TSSs with similar genomic annotations, with 48.7% of the TfR1 peaks mapping to promoters (Fig. 3e). Additionally, through de novo motif discovery, a consensus motif was identified highly enriched in the binding sites of both TfR1 and p53 (Fig. 3f). In addition, we also observed high similarity of known motifs (83.9% overlapped) in both the TfR1 and p53 peaks (Supplementary Fig. 15). Of note, p53 and TfR1 were not only highly relevant in their DNA-binding patterns, but also exhibited a positive correlation in the strength of their peaks (Fig. 3g). To further elucidate the regulatory mechanisms for DNA-binding ability of TfR1 by p53, we performed a comparative analysis of CUT&Tag data between p53-knockdown cells (sh*TP53*) and control cells (sh*SCR*). Consistent with the expectation, the mean intensity of the TfR1 CUT&Tag peaks was reduced in p53-knockdown cells (Fig. 3h). Taken together, these findings suggest that the interaction with p53 endows TfR1 with the potential to regulate gene transcription.

Next, we performed gene ontology (GO) term analysis for the TfR1 peaks in the CUT&Tag data. A series of pathways associated with DNA damage repair were highly enriched, notably highlighting the high ranking of the nucleotide excision repair pathway (Fig. 3i). Six p53 downstream genes related to DNA damage repair, *XPC*, *BBC3*, *CDKN1A*, *DDB2*, *ERCC5*, and *NUFIP2*, from numerous TfR1 peaks, demonstrated distinct TSS binding patterns shared with p53 (Fig. 3j, Supplementary Fig. 16). In summary, the genome-wide analysis suggested that the binding of TfR1 to promoters was functionally relevant to transcriptional regulation, especially for DNA damage repair-related genes.

### Nuclear TfR1 regulates the p53-related NER pathway

**Fig. 4:**
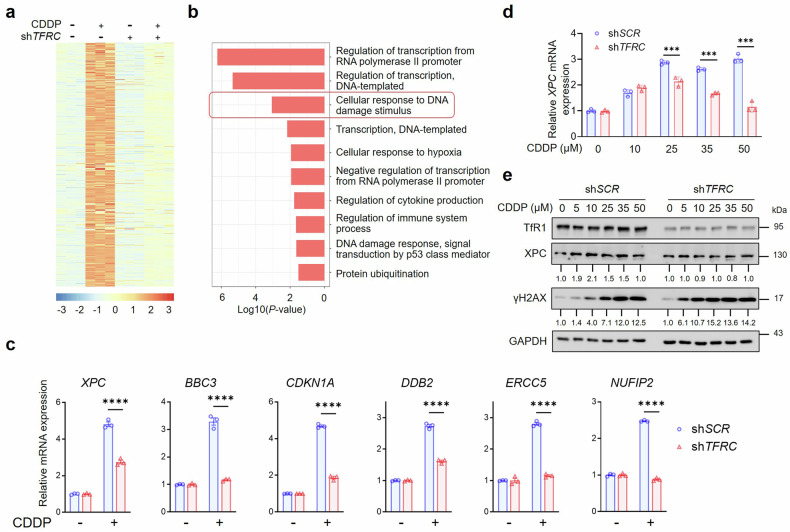
Transcriptomic analysis revealed that nuclear TfR1 regulates the p53-related NER pathway. **a** Heatmap analysis of the top DEGs of sh*TFRC* cells relative to sh*SCR* cells in response to 50 μM CDDP stimulation for 24 h. **b** GO term enrichment analysis of the DEGs of sh*TFRC* cells relative to sh*SCR* cells in response to CDDP stimulation. **c** qPCR analysis of representative DEGs after 50 μM CDDP treatment for 24 h. The *ACTIN* gene was used as an internal control (*n* = 3). **d** qPCR analysis of *XPC* mRNA levels in response to CDDP treatment for 24 h in HCT-116 sh*SCR* or sh*TFRC* cells. The *ACTIN* gene was used as an internal control (*n* = 3). **e** Immunoblot analysis of TfR1, XPC, and γH2AX in response to CDDP treatment for 24 h in HCT-116 sh*SCR* or sh*TFRC* cells. The relative abundance of XPC and γH2AX was normalized to that of the CDDP = 0 controls in sh*SCR* or sh*TFRC* cells. GAPDH was used as a loading control. The data are presented as the mean ± SEM from at least three independent experiments in (**c**) and (**d**). The *P*-value was determined using two-way ANOVA analysis in (**c**) and (**d**). ***P < 0.001; ****P < 0.0001

Subsequently, we performed RNA-seq analysis on TfR1-knockdown cells treated with cisplatin (CDDP), a well-known genotoxic agent that induces DNA damage repair via the NER pathway,^49^ with the aim of identifying differentially expressed genes (DEGs). Intriguingly, the expression of genes whose expression was upregulated by CDDP was attenuated by TfR1 knockdown (Fig. 4a). Functional pathway analysis revealed extremely strong enrichment in the DNA damage response (Fig. 4b). Furthermore, several classical p53 target genes involved in DNA damage repair pathways were further confirmed by qPCR, confirming the pattern observed in the RNA-seq assay (Fig. 4c). A similar result was replicated in Hep G2 cells with wild-type p53 (Supplementary Fig. 17). Notably, since XPC acts as the upstream initiator of the NER pathway,^50^ we focused on investigating the binding of TfR1 to the *XPC* promoter. Through a dual-luciferase reporter assay, we observed that after CDDP stimulation, the upregulation of *XPC* in sh*TFRC* cells significantly decreased compared with that in sh*SCR* cells, demonstrating the transcriptional regulation of *XPC* by TfR1 (Supplementary Fig. 18). In addition, we investigated whether TfR1 regulated XPC expression at both mRNA and protein levels in response to CDDP stimulation. As shown in the qPCR and immunoblot results, the XPC level gradually increased with increasing concentrations of CDDP in HCT-116 cells. In contrast, upon TfR1 depletion, the increasing trend of XPC was significantly impeded (Fig. 4d, e, Supplementary Fig. 19). Additionally, we assessed the phosphorylation of H2AX (γH2AX), a well-established marker of DNA damage. Compared with that in sh*SCR* cells, the depletion of TfR1 led to a significantly higher level of γH2AX. This phenomenon indicated that TfR1 deficiency compromised cellular resistance to CDDP-induced DNA damage, leading to more pronounced γH2AX accumulation (Fig. 4e, Supplementary Fig. 19). In addition, we generated TfR1-knockdown HT-29 cells and MDA-MB-231 cells. These cell lines are known for their loss-of-function p53 mutations, which are theoretically incapable of inducing XPC upregulation in response to DNA damage.^51^ The immunoblot analysis revealed no change in the regulation of XPC or γH2AX in cell lines with p53 mutations, independent of TfR1 status (Supplementary Fig. 20a, b). Overall, these experiments suggest that nuclear TfR1 exhibits a profound influence on the expression of p53 downstream NER-related genes.

### Cellular function demonstrates the role of nuclear TfR1 in tumor progression in response to DNA damage

**Fig. 5:**
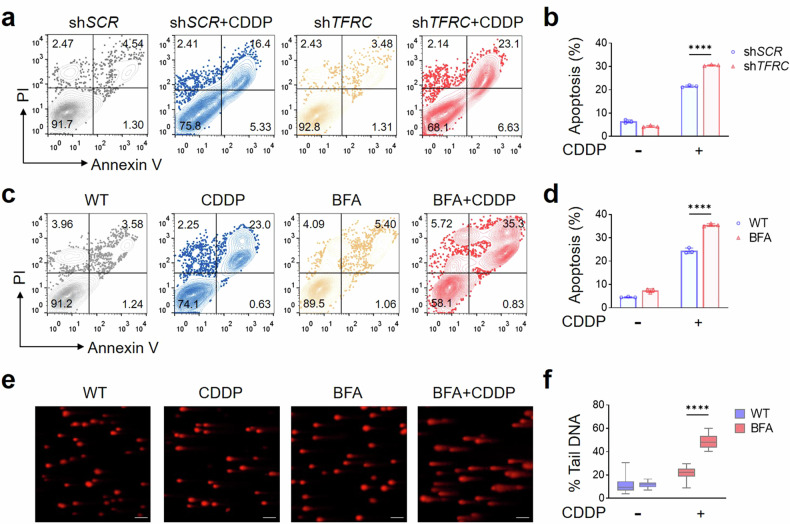
Cellular function demonstrates the role of nuclear TfR1 in tumor progression in response to DNA damage. **a** Assessment of apoptosis in HCT-116 sh*SCR* or sh*TFRC* cells after 50 μM CDDP treatment for 24 h. **b** Quantification of the percentage of apoptotic cells (**a**) (*n* = 3). **c** Assessment of apoptosis in response to 50 μM CDDP stimulation for 24 h with or without pretreatment with 500 nM BFA for 6 h. **d** Quantification of the percentage of apoptotic cells in (**c**) (*n* = 3). **e** Representative images of the alkaline comet assay after 1 μM CDDP treatment for 6 h with or without pretreatment with 500 nM BFA for 6 h. Scale bar = 100 μm. **f** Quantification of the percentage of DNA in the comet tail (% Tail DNA) of (**e**) (*n* = 50). The data were presented as the means ± SEMs from at least three independent experiments in (**b**, **d**, and **f**). The *P*-value was determined using two-way ANOVA analysis in (**b**, **d**, and **f**). ****P < 0.0001

We then assessed the pathological importance of nuclear TfR1 in tumor progression. As shown in Supplementary Fig. 21a, b, knockdown of TfR1 led to a 20% reduction of IC_50_, suggesting increased susceptibility to CDDP treatment. A rescue experiment was further performed in TfR1-knockdown cells via re-expression of a short hairpin RNA (shRNA)-resistant form of TfR1 (RES-TfR1-KD), which restored CDDP resistance, whereas cells transfected with an empty vector (EV-TfR1-KD) did not exhibit restored resistance. Given that CDDP is a well‐established apoptosis inducer,^52^ we observed a notable increase in the percentage of apoptotic cells, with a 1.42-fold elevation upon CDDP treatment in TfR1 knockdown cells compared to control cells (Fig. 5a, b).

Moreover, by utilizing BFA treatment to significantly impede the nuclear import of TfR1 without affecting the overall TfR1 level, we observed enhanced sensitivity to CDDP. This was evident from a substantial elevation in apoptosis compared to CDDP treatment alone (Fig. 5c, d). Next, we performed a comet assay, which is a sensitive technique for detecting DNA damage at the individual cell level. As presented in Fig. 5e, f, reducing the proportion of nuclear TfR1 via BFA treatment resulted in increased accumulation of CDDP-induced DNA breaks. These findings collectively emphasized that TfR1 nuclear translocation is important for tumor progression in vitro in response to DNA damage.

Conversely, we evaluated the sensitivity of the aforementioned TfR1-knockdown HT-29 cells and MDA-MB-231 cells harboring loss-of-function mutant p53 to CDDP compared with that of HCT-116 cells expressing functional p53. As expected, the IC_50_ remained unchanged in these two cell lines after TfR1 knockdown (Supplementary Fig. 22). These results suggest that nuclear TfR1 modulates the NER pathway in a p53-dependent manner. Furthermore, these experiments suggest that the variability in the function of nuclear TfR1 in gene regulation is based on alterations in p53 status.

### Phenomics elucidates the clinical relevance of nuclear TfR1 with tumor progression

**Fig. 6:**
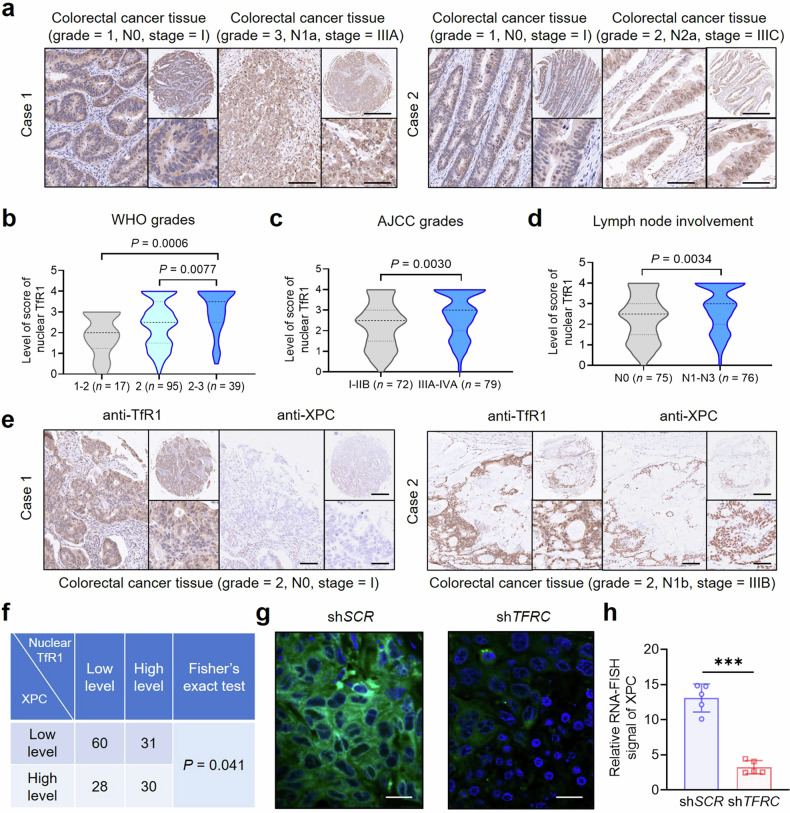
Influence of nuclear TfR1 on tumor progression. **a** Representative 2 cases of IHC images (×2, ×10, and ×20 magnifications) from the CRC tissue microarray (*n* = 151). Scale bar = 500 μm (magnification ×2); scale bar = 100 μm (magnification ×10); scale bar = 50 μm (magnification ×20). **b**–**d** Correlations between the nuclear TfR1 level score and WHO grade (**b**), AJCC stage (**c**), and lymph node involvement (**d**) in CRC samples. **e** Representative 2 cases of IHC images (×2, ×10, and ×20 magnifications) of TfR1 and XPC staining in the CRC tissue microarray. Scale bar = 500 μm (magnification ×2); scale bar = 100 μm (magnification ×10); scale bar = 50 μm (magnification ×20). **f** Chi-square test of the correlation between the levels of nuclear TfR1 and XPC determined via IHC staining. **g** Representative images from RNA-FISH analysis showing *XPC* mRNA levels in HCT-116 sh*SCR-* or sh*TFRC*-xenografted tumor tissues (*n* = 5). CDDP (1 mg/kg) was intratumorally injected into the tumors 24 h before harvest. Nuclei were stained with DAPI. Scale bar = 10 µm. **h** Quantification of the relative fluorescence intensity of RNA-FISH images. The data were presented as the means ± SEMs of at least three independent experiments in (**h**). *P*-values were determined via one-way ANOVA in (**b**), via unpaired two-tailed Student’s *t*-tests in (**c**, **d**, and **h**), and via chi-square tests and Fisher’s exact tests in (**f**). ***P < 0.001

The clinical relevance of nuclear TfR1 in tumor progression was explored after elucidating the molecular mechanisms in regulating the DNA damage repair pathway. According to the GEPIA database, the discrepancy in TfR1 expression between CRC, including colon adenocarcinoma (COAD) and rectum adenocarcinoma (READ), and normal tissues was notably substantial, indicating that it was one of the most significant differences observed (Supplementary Fig. 23a, b). We subsequently confirmed the upregulation of TfR1 in tumor tissue sections from clinical primary CRC patients compared with normal tissue (Supplementary Fig. 24). Consequently, we postulated that TfR1 may play an important role in CRC progression. To further evaluate the clinical relevance of nuclear TfR1, we performed IHC analysis using CRC tissue microarrays (Fig. 6a, Supplementary Table 1). Strikingly, our findings demonstrated a positive correlation between nuclear TfR1 levels and various clinical indicators. Analysis based on WHO (World Health Organization) grades revealed that the increased nuclear TfR1 level was correlated with an increased degree of tumor malignancy (Fig. 6b), as further evidenced by its positive association with the Ki-67 level (Supplementary Fig. 25a, b). Moreover, the nuclear TfR1 level was positively related to the American Joint Committee on Cancer stage, demonstrating its ability to facilitate aggressive features (Fig. 6c). In addition, the association with lymph node involvement revealed the positive role of nuclear TfR1 in metastasis (Fig. 6d). These results unveiled the positive impact of nuclear TfR1 on tumor progression.

To further validate the regulatory effect of nuclear TfR1 on the NER pathway in vivo, we detected the XPC level in the same CRC clinical samples through IHC analysis. Consistently, nuclear TfR1 staining was positively correlated with XPC staining (Fig. 6e). Chi-square analysis confirmed a significant correlation between the nuclear TfR1 level and XPC expression (Fig. 6f). To validate in vivo XPC expression in response to DNA damage influenced by nuclear TfR1, we constructed a TfR1-knockdown xenograft tumor model following CDDP administration. As shown by fluorescence RNA in situ hybridization (FISH) analysis, the mRNA expression of *XPC* in sh*TFRC* tumors was lower than that in sh*SCR* tumors (Fig. 6g). The quantification of the relative RNA-FISH signal indicated a statistically significant difference (Fig. 6h). In summary, our findings confirmed the pathological association between nuclear TfR1 and XPC, emphasizing the potential clinical relevance of TfR1 nuclear translocation in the NER pathway.

## Discussion

**Fig. 7:**
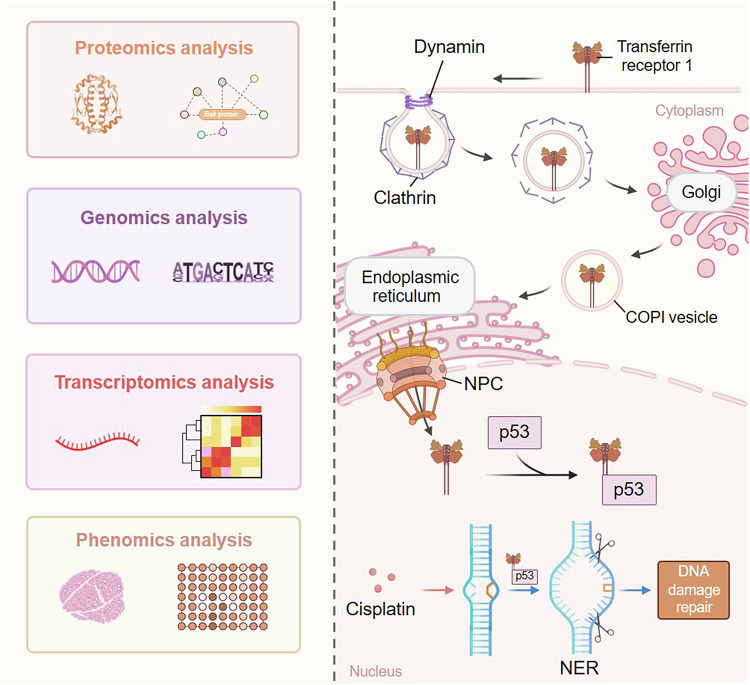
Schematic model showing the nuclear translocation route and functional role of nuclear TfR1. TfR1 is first dissociated from the plasma membrane by clathrin-mediated endocytosis. Next, it is transported in the form of vesicles to the perinuclear region via the Golgi complex–ER retrotranslocation pathway and is imported through the nuclear pore complex. Through multi-omics analysis, we elucidated that once TfR1 enters the nucleus, it interacts with p53 and positively regulates the transcription of p53 downstream genes, especially those in the NER pathway, thereby promoting tumor progression. This figure was created with BioRender (https://biorender.com)

Over the past few decades, most studies on TfR1 have focused primarily on its classical function in mediating iron uptake. In the context of cancer, the iron uptake function is widely regarded as the primary mechanism through which TfR1 promotes tumor development. Recently, a growing body of evidence has emphasized the significance of TfR1 in multiple non-classical cellular pathways. Nevertheless, a pressing issue is that the precise molecular mechanisms are less well defined. The unexpected identification of TfR1 nuclear localization in our prior study provides a promising opportunity for us to revisit this question. Considering that the function of protein is largely determined by subcellular localization, investigations of the nuclear localization of TfR1 may provide new insight. In this work, we demonstrated that nucleus-localized TfR1 interacts with p53 and performs novel functions by regulating gene expression. This interaction significantly augments the DNA damage repair capacity, thereby promoting tumor progression (Fig. 7).

Although we previously observed TfR1 in the nucleus, this phenomenon was not confirmed. Here, we systematically confirmed the nuclear localization and translocation pathway of TfR1 via various experiments both in vitro and in vivo, including IHC analysis, subcellular fractionation followed by immunoblotting, and confocal microscopy. Our findings provide support for a model: the nuclear transport of TfR1 initiates with clathrin-mediated endocytosis at the plasma membrane and is followed by the Golgi complex-coatomer protein I-coated vesicle-ER retrograde trafficking pathway to reach the perinuclear region. Given that various RTK receptors have been verified to follow this trafficking pathway, it is reasonable to infer that TfR1 also utilizes this route, especially considering its structural similarity—the homodimeric configuration—shared with RTK family receptors. Upon reaching the perinuclear region, many receptors require the assistance of nuclear transporters to traverse the nuclear membrane. For example, importin-α is reported to facilitate the nuclear translocation of the type 1 parathyroid hormone receptor.^53^ Likewise, importin-β1 mediates the nuclear import of coagulation factor II receptor-like 1 across the nuclear pore complex,^54^ whereas transportin-1 has been implicated in the nuclear trafficking of CD44.^55^ In this study, we revealed that TfR1 interacts with all three of these nuclear transporters, suggesting a potential mechanism by which TfR1 is translocated into the nucleus. However, we did not observe the inhibition of TfR1 nuclear import after knocking down any one of these three nuclear transporters (data not shown). Therefore, the specific molecular mechanism of TfR1 translocation around the nuclear pore complex deserves further investigation. Another important question to be addressed is the identification of the critical region responsible for the nuclear import of TfR1. Our current findings indicate that the ICD of TfR1 is not essential for its nuclear translocation. However, further investigations are required to elucidate the underlying mechanism in future studies.

The next critical question is: How can nuclear TfR1 perform its non-classical biological function in the absence of a canonical DNA-binding domain? One plausible hypothesis is that this process is substantially mediated via the interactions with other nuclear proteins. To investigate this possibility, we conducted mass spectrometry to identify potential TfR1 interactors and identified p53 as a top-ranked candidate. This interaction between TfR1 and p53 was subsequently validated both in vitro (in tumor cell lines) and in vivo (in xenograft tumor tissues in animal models). Further verification using BIAcore revealed a direct interaction between TfR1 and p53. Co-IP analysis involving p53 truncations and point mutants revealed that amino acids 51–53 of p53 are crucial for binding to TfR1, suggesting that p53 serves as a crucial functional mediator of nuclear TfR1 activity. Consistent with these findings, our genomic data revealed that TfR1 predominantly binds to promoter regions, with a notable enrichment of specific nucleotide sequences. Importantly, the genome-wide binding profiles of nuclear TfR1 and p53 exhibit striking similarities, both in terms of binding loci and binding intensities. Moreover, knockdown of p53 significantly diminished the genome-wide binding of TfR1, further supporting the conclusion that p53 facilitates the nuclear functions of TfR1. Collectively, these findings offer a compelling mechanistic explanation for the potential of TfR1 to execute novel functions within the nucleus.

The DNA damage repair-related pathway has been recognized as a promising therapeutic target in CRC, and the drug development efforts in this field have progressed into the early stages of clinical translation.^56^ Interestingly, another critical feature of the CUT&Tag data and the RNA-seq data is the considerable enrichment of nuclear TfR1 target genes in the DNA damage repair-related pathway, especially the NER pathway. Notably, the NER pathway is one of the major mechanisms by which p53 modulates DNA damage repair capacity.^57,58^ More importantly, nuclear TfR1 is able to positively regulate the expression of these genes, such as *XPC*, *DDB2*, and *ERCC5*. As a result, TfR1 nuclear translocation significantly enhances the resistance of tumor cells to DNA damage, as demonstrated by cell apoptosis assays, cell viability assays, and comet assays. Among the NER-related genes, *XPC* has emerged as particularly noteworthy due to its pivotal role in initiating DNA damage recognition. Importantly, the clinical relevance of *XPC* in CRC has been reported. Elevated XPC expression in clinical samples correlates with increased malignancy, whereas knockdown of XPC markedly suppresses tumor growth in the murine xenograft model.^59^ Intriguingly, we revealed a significant correlation between the nuclear TfR1 level and XPC expression in clinical specimens via IHC analysis. In addition, the elevated nuclear TfR1 level is significantly correlated with increased malignancy, aggressive features, and metastasis in many clinical tumor samples. Collectively, these results reveal a novel mechanism by which carcinoma cells may exploit nuclear TfR1 to facilitate their survival and progression in the context of DNA damage.

In conclusion, this work illustrates that TfR1 not only acts as a downstream “gatekeeper” predominantly involved in iron uptake but also plays a role as an upstream “commander” for the regulation of gene expression. While we have focused primarily on the NER pathway, it is worth emphasizing that nuclear TfR1 has the potential to regulate other facets of cellular signaling pathways. Future studies on these pathways will pave the way for deciphering the unidentified identities of TfR1, thereby facilitating the development of TfR1 as a key therapeutic target in the context of tumor treatment.

## Materials and methods

### Cell culture

All cell lines were obtained from the American Type Culture Collection, except for MX-1 cells (a TfR1-negative cell line)^60^ and HCT-116-p53^−^^/^^−^ cells, which were obtained from the cell bank of the Committee on Type Culture Collection of the Chinese Academy of Sciences and Fenghui Biotechnology Co., Ltd. (China). HCT-116 and A549 cells were cultured in RPMI-1640 medium supplemented with 10% FBS. HT-29, MDA-MB-231, U-87 MG, MX-1, and HEK293T cells were cultured in DMEM medium supplemented with 10% FBS. Hep G2 cells were cultured in MEM medium supplemented with 10% FBS, 1% non-essential amino acids, and 1% sodium pyruvate. HEC-1B cells were cultured in EMEM medium supplemented with 10% FBS. All culture media were supplemented with 1% penicillin‒streptomycin. All the cells were cultured at 37 °C in an atmosphere with 5% CO_2_, and the culture media were replaced every 2 days.

### Animals

Female BALB/c nude mice (6–8 weeks old) were purchased from Gempharmatech Co., Ltd. (China). All animal studies were performed according to the protocol approved by the Institutional Animal Care and Use Committee at the Institute of Biophysics, Chinese Academy of Sciences (SYXK2024210). For xenograft tumor model construction, approximately 1.2 × 10^7^ HCT-116 cells were injected subcutaneously into BALB/c nude mice. For the Co-IP experiments, when the tumor diameter had reached 0.4–0.6 cm, the tumors were excised, homogenized with a tissue homogenizer and lysed with RIPA lysis buffer. For the RNA-FISH experiment, once tumors were detectable, the mice were randomized and intratumorally injected with sh*SCR* or sh*TFRC* lentivirus particles (MOI = 30) every 2 days. After 10 days, the mice were intratumorally injected with CDDP and sacrificed, after which the tumors were harvested, fixed, paraffin-embedded, and sectioned.

### Plasmids

To generate Flag-TfR1- and TfR1-EGFP-expression plasmids, human TfR1 cDNA (NCBI gene ID: 7037) was subcloned and inserted into the pQCXIH vector, which was tagged with 3×Flag at the N-terminus or EGFP at the C-terminus. To generate TfR1-SPSS expression plasmids, human TfR1 cDNA with a mutation in amino acids 58–61 (from KPKR to SPSS) was subcloned and inserted into the pQCXIH vector, which was tagged with 3×Flag at the N-terminus. For the construction of TfR1 truncation plasmids, human TfR1 cDNA with a deletion of amino acids 1–67 was subcloned and inserted into the pQCXIH vector, which was tagged with 3×Flag at the N-terminus, wherein the sites of the truncations were referred to the classification of the TfR1 domains.^61^ To generate p53 truncation plasmids, human p53 cDNA with a deletion at amino acids 1–47, 48–62, 1–62, 1–50, or 51–53 was subcloned and inserted into the pCDH vector, wherein sites of truncations was referred to the classification of p53 transactivation domain 1 (TAD1) and transactivation domain 2 (TAD2).^62^ To generate p53-E51A + Q52A + W53A mutant plasmids, human p53 cDNA with point mutations at amino acids 51–53 (changing EQW to AAA) was subcloned and inserted into the pCDH vector. To generate TfR1 expression plasmids for the rescue experiment, human TfR1 cDNA, which was code-optimized in a shRNA-resistant form, was subcloned and inserted into the pQCXIH vector. To generate TfR1-knockdown (sh*TFRC*), p53-knockdown (sh*TP53*), and scrambled shRNA (sh*SCR*) plasmids, *TFRC*-targeted shRNA (sense strand sequence: 5’-GCCCAGATGTTCTCAGATATTT-3’), *TP53*-targeted shRNA (sense strand sequence: 5’-CGGCGCACAGAGGAAGAGAATCTC-3’), or scrambled sequence were subcloned and inserted into the PLKO.1-puro vector. To generate the *XPC* promoter reporter plasmid, the human *XPC* promoter was subcloned and inserted into the pGL3-Basic vector. To generate the ectodomain of TfR1 expression plasmids, human TfR1 cDNA (encoding TfR1 residues 88–760) was synthesized and inserted into pcDNA3.1(+) with the Kozak sequence (GCCACC) and the signal peptide from albumin fused at the N-terminus. A human rhinovirus 3C (HRV3C) protease recognition sequence, an octa-histidine tag and a strep-tactin tag II were placed at the C-terminus. To generate p53 expression plasmids, human p53 cDNA (NCBI gene ID: 7157) optimized for the *Escherichia coli* codon was subcloned and inserted into the pET30a vector. An octa-histidine tag and a strep-tactin tag II were also placed at the C-terminus.

### Antibodies and chemicals

All antibodies used in this study, except the anti-TfR1 antibody (clone number: 23B10), were purchased from commercial companies. These antibodies include anti-TfR1 antibody (Sigma, HPA028598), anti-Lamin B1 antibody (Abcam, ab133741), anti-α-Tubulin antibody (Beyotime, AT819), anti-Calreticulin antibody (Abcam, ab92516), anti-Syntaxin-6 antibody (CST, 2869), anti-importin-β1 antibody (Abcam, ab2811), anti-importin-α antibody (Abcam, ab307438), anti-Transportin-1 antibody (CST, 31452), anti-Sec61β antibody (CST, 14648), anti-GAPDH antibody (ABclonal, AC033), anti-Flag antibody (Sigma, F3165), anti-p53 antibody (Santa Cruz, sc-126), anti-p53 antibody (CST, 2524), anti-p53 antibody (Abcam, ab26), anti-XPC antibody (Santa Cruz, sc-74410), anti-γH2AX antibody (Abcam, ab81299), anti-Ki67 antibody (CST, 9449), mouse IgG (CST, 5415), rabbit IgG (CST, 3900), anti-RS4X antibody (Immunoway, YT4135). The 23B10 anti-TfR1 antibody was prepared in BALB/c mice using a standard protocol of hybridoma technology.^63^

The chemicals used in this study include: chlorpromazine hydrochloride (Macklin, C834105), brefeldin A (Sigma, B5936), cis-platin (Sigma, P4394), polybrene (Yeasen, 40804ES76), hygromycin B (Yeasen, 60224ES03), and puromycin (Yeasen, 60209ES10).

### Retrovirus and lentivirus infection

To generate retroviruses, HEK293T cells were transfected with Flag-TfR1-, TfR1-EGFP-, or shRNA-resistant TfR1-expression plasmids and co-transfected with helper plasmids (pMD-VSV-G and pMD-gag-pol). For the generation of lentiviral particles, HEK293T cells were transfected with sh*SCR*, sh*TFRC*, or sh*TP53* plasmids and co-transfected with helper plasmids (pMDLg/pRRE, pCMV-VSV-G, and pRSV-Rev). Transfections were performed using Fugene HD transfection reagent (Promega, E2311). After 48 h, the retroviral or lentiviral supernatants were collected, filtered through 0.45 μm filters, and used to infect the indicated cells supplemented with polybrene (10 μg/mL). After retroviral or lentiviral infection for 36–48 h, the cells were selected with hygromycin (250 μg/mL) or puromycin (1 μg/mL).

### siRNA transfection

For the knockdown of Sec61β, HCT-116 cells were transfected with a scrambled sequence siRNA or a mixture of 2 different siRNA sequences targeting *SEC61B* (5’-GCAAGUACACUCGUUCGUA-3’ and 5’-GCAAGUACACUCGUUCGUA-3’) using Lipofectamine RNAi MAX (Invitrogen, 13778150) for 72 h.

### Immunohistochemistry

The tissue microarray sections were incubated with anti-TfR1 antibody (23B10) or anti-XPC antibody overnight at 4 °C, followed by incubation with horseradish peroxidase (HRP)-conjugated goat anti-mouse secondary antibody (ZSBG-BIO, PV-6002) for 30 min at 37 °C. Diaminobenzidine (ZSBG-BIO, ZLI-9019) and hematoxylin (ZSBG-BIO, ZLI-9610) were used for staining. Images were obtained using an Aperio CS2 slide scanner (Leica Biosystems, Germany). Two independent pathologists who were blinded to all the clinical information scored the samples. Nuclear TfR1 and XPC levels were calculated as the product of a proportion score and an intensity score. The proportion score reflected the fraction of positively stained cells (score 0, <5%; score 1, 5–10%; score 2, 10–50%; score 3, 50–75%; score 4, >75%). The intensity score represented the staining intensity (score 0, negative; score 1, weak; score 2, moderate; score 3, strong). The total score was calculated by the multiplication of the proportional score and intensity score and was further divided into 5 levels as follows: level 0 = 0, level 1 = 1–3, level 2 = 4–6, level 3 = 7–9, and level 4 = 10–12.

### Human tissue microarray

The study using the tissue microarray was approved by the Life Sciences Ethics Committee of Changsha Yaxiang Biotechnology Co., Ltd. (China). The ethics report is available online at yxswll.ccrl.cn. The query code is Csyayj2024017. The human multi-organ cancer tissue microarray X234Mc01, human colorectal cancer tissue microarray D1060401 and D062Co01 were obtained from Bioaitech Co., Ltd. (China). The multi-organ cancer tissue microarray X234Mc01 included 204 cancerous samples. The tissue microarray D1060401 included 95 cancerous samples from 61 male and 34 female patients with ages ranging from 22 to 78 years (mean age of 55). As for the American Joint Committee on Cancer grade, there were 15 cases of Stage I patients, 19 cases of Stage IIA patients, 12 cases of Stage IIB patients, 5 cases of Stage IIIA patients, 31 cases of Stage IIIB patients, 9 cases of Stage IIIC patients, 2 cases of Stage IVA patients, and 2 cases of Stage IVB patients. As for WHO (World Health Organization) grade, there were 10 cases of grade 1 patients, 7 cases of grade 1–2 patients, 59 cases of grade 2 patients, 3 cases of grade 2–3 patients, and 16 cases of grade 3 patients. As for lymph node involvement, there were 48 cases of patients without lymph node involvement, and 47 cases of patients with lymph node involvement. The tissue microarray D062Co01 included 56 cancerous samples from 33 male and 24 female patients with ages ranging from 39 to 84 years (mean age of 58). As for American Joint Committee on Cancer grade, there were 5 Stage I patients, 14 Stage IIA patients, 7 Stage IIB patients, 1 Stage IIIA patient, 20 Stage IIIB patients, 7 Stage IIIC patients, 1 Stage IVA patient, and 1 Stage IVC patient. With respect to WHO grade, there were 36 grade 2 patients, 5 grade 2–3 patients, and 15 grade 3 patients. As for lymph node involvement, there were 27 patients without lymph node involvement and 29 patients with lymph node involvement.

### Confocal microscopy

Various types of tumor cells were seeded on glass-bottom cell culture dishes. After 24 h, the cells were fixed with 4% paraformaldehyde for 15 min and permeabilized with 0.2% Triton X-100 for 20 min. Then, the cells were blocked with 10% goat serum for 1 h and incubated with anti-TfR1 antibody (Sigma, HPA028598) or rabbit IgG overnight at 4 °C. Finally, the cells were incubated with Goat anti Rabbit IgG (H+L) cross-adsorbed secondary antibody Alexa Fluor^TM^ 647 (A21244, Invitrogen) for 1 h and stained with DAPI (Solarbio, S2110) for 10 min at room temperature in the dark. The TfR1-EGFP expression HCT-116 cells was imaged after staining with Hoechst (Beyotime, C1028) for 10 min at room temperature in the dark. Images were captured using a laser scanning confocal microscope (ZIESS-LSM700, German).

### Subcellular fractionation

To isolate the nuclei, 1–2 × 10^6^ cells were resuspended in the nuclear isolation buffer composed of 10 mM HEPES, pH 7.9, containing 10 mM KCl, 1.5 mM MgCl_2_, 0.34 M sucrose, 10% glycerol, 1 mM dithiothreitol, 1 mM phenylmethanesulfonyl fluoride, and 0.1% Triton X-100. After that, the cells were incubated on ice for 15 min and centrifuged at 1300×*g* for 5 min at 4 °C. The pellet was washed with nuclear isolation buffer (without Triton X-100) and collected as the nuclear fraction. The supernatant was further centrifuged at 1700×*g* for 5 min at 4 °C to obtain the non-nuclear fraction.

### Immunoblotting

The protein concentration was determined using the BCA reagent (Thermo Scientific, 23228). The proteins were subsequently boiled with SDS loading buffer, separated on 10–12% SDS-PAGE gels, and transferred to polyvinylidene difluoride membranes. The membranes were blocked with nonfat milk (5% in PBS) and incubated with the indicated primary antibodies, followed by incubation with an HRP-conjugated goat anti-rabbit IgG (H+L) secondary antibody (Emarbio, EM35111) or an HRP-conjugated goat anti-mouse IgG (H+L) secondary antibody (Emarbio, EM35110). Finally, the proteins were detected via an enhanced chemiluminescence (ECL) system (Touch imager, E-blot, Shanghai, China).

### Immunoprecipitation

Various types of tumor cell lines were harvested from 100 mm dishes. To detect the interaction between TfR1 and p53, the cells were lysed and the nuclear fraction was isolated as described previously, and anti-TfR1 antibody (Sigma, HPA028598) was used as the immunoprecipitation part. When detecting the specificity of the 23B10 antibody to recognize human TfR1, the 23B10 antibody, other clones (24D5 and 8F10) produced from the same batch, and the anti-TfR1 antibody (Sigma, HPA028598) were used as the immunoprecipitation part and the whole cell lysate was obtained using cell lysis buffer (Sigma, C2978) supplemented with 1 mM phenylmethanesulfonyl fluoride and phosphatase inhibitor cocktail (Beyotime, P1081) for 1 h at 4 °C. Then, the lysates were subsequently incubated with protein G beads (Santa Cruz, sc-2002) overnight at 4 °C. When validating the interactions between TfR1 and importin-β, importin-α, transportin-1, or Sec61β, anti-Flag antibody (Sigma, F3165) was used as the immunoprecipitation part and the whole cell lysates were obtained using cell lysis buffer as described previously, followed by incubation with anti-Flag antibody-conjugated beads overnight at 4 °C. All the beads were subsequently washed with cell lysis buffer (supplemented with 250 mM NaCl) 3 times. After that, the protein G beads were boiled with 1×SDS loading buffer. The anti-Flag antibody-conjugated beads were eluted with 3×Flag peptide for 4 h at 4 °C, and the supernatants were boiled with 1×SDS loading buffer.

### Mass spectrometry analysis

The proteins pulled down from immunoprecipitation were separated with silver-dyed gels. Then, the silver-dyed gels were excised, decolorized, reduced with dithiothreitol, alkylated with iodoacetamide, and digested with trypsin overnight to obtain the peptide fragments. The peptide fragments were extracted with 60% CAN and were analyzed by Liquid Chromatography-Mass Spectrometry/Mass Spectrometry (LC-MS/MS) on NanoLC-Q EXACTIVE (Thermo Scientific, USA). Proteins were identified by searching the fragment spectra in the UniProt_proteome_human_2018 database.

### Expression and purification of recombinant human TfR1 and p53

For human TfR1 expression and purification, the ectodomain of TfR1 expression plasmids was transiently transfected into HEK293T cells using Lipofectamine 2000 (Thermo Scientific, 11668500) as the transfection agent. Fresh medium was replaced after 7 h of transfection. After another 96 h, the supernatants were harvested, and TfR1 was purified via a Ni-NTA-affinity column (Cytiva, USA) and a strep-tactin column (IBA Biotagnology, Germany), followed by HRV3C protease cleavage to remove the affinity tags. After HRV3C protease cleavage, the protein sample was purified with a Ni-NTA column to remove the affinity tag and HRV3C protease. The protein sample from the previous Ni-NTA-affinity column was further polished with a Superdex 200 Increase 10/300 GL (Cytiva, USA) column to remove protein aggregates and trace amounts of miscellaneous proteins. The purified TfR1 protein was stored at −80 °C in 10 mM HEPES (pH 7.5) with 150 mM NaCl. The quality and quantity of the purified protein were evaluated by SDS-PAGE and UV/visible spectra using the theoretical ε280 nm 96,260 M^−^^1^ cm^−^^1^. For human p53 expression and purification, *E. coli* BL21 (DE3) cells were transformed with p53 expression plasmids and was grown to OD600 0.6 at 37 °C in 0.8 L of kanamycin-containing 2-YT medium. Gene expression was induced by the addition of 0.4 mM isopropyl-1-thio-β-D-galactopyranoside, and the cells were further grown at 16 °C for 20 h. After cell harvesting, the pellet was suspended in 50 mM Tris-HCl (pH 8.0) with 10 mM 2-mercaptoethanol, 5 mM imidazole and 250 mM NaCl and was disrupted by high-pressure cracker in the presence of 1 mM phenylmethanesulfonyl fluoride. The lysate was centrifuged, and the supernatant was loaded onto the Ni-NTA-affinity column. The eluent from the Ni-affinity column was further purified with the strep-tactin column. The p53 protein from the strep-tactin column was concentrated, and the buffer was changed to 10 mM HEPES (pH 7.5) with 100 mM NaCl and 1 mM DTT using a Hitrap Desalting column (Cytiva, USA) and stored at −80 °C. The quality and quantity of the purified protein were evaluated by SDS-PAGE and UV/visible spectroscopy via the theoretical ε280 nm 40,910 M^−^^1^ cm^−^^1^.

### Surface plasmon resonance

The recombinant human TfR1 was further biotinylated and immobilized on a streptavidin-coated chip. The binding affinity measurements were performed at 25 °C in PBS (pH 7.4) supplemented with 0.05% Tween-20 via a BIAcore T100 surface plasmon resonance instrument (GE Healthcare, USA). Approximately 150 response units of biotinylated TfR1 were captured in flow cells 2. Single-cycle kinetics were carried out by injecting increasing concentrations (25, 50, 100, 200, and 400 nM) of recombinant human p53, which flowed over Flow cell 1 and Flow cell 2. Binding responses for kinetic analysis were blank- and reference-subtracted. The binding curve was fit with a 1:1 binding model using BIAcore Instrument software.

### CUT&Tag assay

The CUT&Tag assay was performed with the Hyperactive Universal CUT&Tag Assay Kit from Illumina (Vazyme Biotech, TD903). For each sample, 1 × 10^5^ cells were harvested, and the nuclei were isolated with nuclear extraction buffer according to the manufacturer’s instructions. The nuclear lysates were incubated with anti-TfR1 antibody (Sigma, HPA028598), anti-p53 antibody (CST, 2524), mouse IgG, or rabbit IgG. Following the manufacturer’s instructions, CUT&Tag libraries were obtained and sequenced by Berry Genomics. The data were analyzed with IGV_2.11.1. CUT&Tag sequencing reads were aligned to the human hg38 genome using Bowtie2 (v2.2.5)^64^ with the parameters: -t -q -N 1 -L 25 after trimming adapters by Trim Galore (v0.6.6). Multiple mapped reads and PCR duplicates were removed to generate uniquely mapped reads via JAVA Picard MarkDuplicates (v2.26.6). All unique mapped reads were then normalized by calculating the number of reads per kilobase per million of sequenced reads for downstream analysis using deepTools bamCoverage (v3.5.0)^65^ with the parameters --binSize 100 -- normalize using reads per kilobase per million. Heatmaps of genome peaks and the distribution of unique peaks were generated with deepTools. All the genome peaks were identified via MACS2 (v2.2.7.1)^66^ with the parameters -nomodel -q 0.05 -g hs. Peaks were compared by BEDTools (v2.29.2).^67^ The Pearson correlation coefficient was used to compare the relevance between different samples, and the significance of the relevance was based on the *P*-value. ChIPseeker (v1.26.2) was used to identify and visualize the features of the peaks.^68^ Promoters were defined as ±2.5 kb around the transcription TSSs. GREAT tool was used to analyze GO enrichment for unique peaks.^69^ The features of DNA motif enrichment of the genome peaks were analyzed by HOMER.^70^

### Cell viability assay

Cell viability was determined using the CCK8 assay. Cells (16,000 cells per well for HCT-116 cells and HT-29 cells, and 10,000 cells for MDA-MB-231 cells after stable transfection with sh*SCR* or sh*TFRC*, respectively) were seeded in 96-well plates overnight. After CDDP treatment, CCK8 solution (Dojindo, CK04) was added into each well at a volume ratio of 10%. The absorbance at 450 nm was measured with a microplate spectrophotometer (SpectraMax M4, Molecular Devices, USA). The half maximal inhibitory concentration (IC_50_) values were calculated with GraphPad Prism 7.

### Flow cytometry analysis

The cell apoptosis assay was performed with flow cytometry using the Annexin V-FITC Apoptosis Detection Kit (Dojindo, AD10). The cells (375,000 cells per well for HCT-116 cells or HCT-116 cells following stable transfection with sh*SCR* or sh*TFRC*) were seeded in 12-well plates overnight. After CDDP or BFA treatment, the cells were resuspended in Annexin V binding solution and stained with Annexin V-FITC and propidium iodide for 15 min at room temperature in the absence of light. After staining, the cells were washed and resuspended in Annexin V binding solution. For the validation of the specific binding to human TfR1 using the anti-TfR1 antibody 23B10, HCT-116 cells stably transfected with sh*SCR* or sh*TFRC* were seeded in 12-well plates overnight (375,000 cells per well). Subsequently, the cells were harvested, fixed with the Fixation Buffer (Biolegend, 420801), permeabilized with the Intracellular Staining Perm Wash Buffer (Biolegend, 421002), and incubated with the anti-TfR1 antibody 23B10 for 1 h at 4 °C. After that, the cells were washed with the Intracellular Staining Perm Wash Buffer and incubated with the goat anti-mouse IgG (H+L) cross-adsorbed secondary antibody Alexa Fluor^TM^ 488 (Invitrogen, A11001) for 30 min at room temperature in the dark. After staining, the cells were washed and resuspended in PBS. The fluorescence signals from at least 10,000 cells were collected with a FACS Calibur (BD Biosciences, USA). The data were analyzed with FlowJo_V10.

### Alkaline comet assay

Alkaline comet assay was performed using the DNA Damage Detection Kit (Keygentec, KGA240). Cells (375,000 cells per well for HCT-116 cells) were seeded in 12-well plates overnight. After CDDP treatment, cells in each well were harvested, washed with PBS, and added to frosted microscope slides covered with normal melting agarose and low melting agarose (10,000 cells/slide) according to the manufacturer’s instructions. Briefly, cells on the slides were lysed with lysis buffer for 1.5 h at 4 °C and incubated in alkaline electrophoresis buffer (1 mmol/L EDTA, 300 mmol/L NaOH) for 40 min for DNA unwinding under an ice bath. Electrophoresis was performed at 25 V for 25 min. Then, the slides were washed with 0.4 mM Tris-HCl (pH 7.5) at 4 °C three times and stained with propidium iodide for 10 min at room temperature in the dark. The slides were visualized using a laser scanning confocal microscope (ZIESS-LSM700, German). At least 50 cells were counted per group. DNA in the tail (% Tail DNA) from the images was analyzed by CaspLab-Comet Assay Software Project (v1.2.3).

### RNA-seq

HCT-116 cells after stable transfection with sh*SCR* or sh*TFRC* were seeded in 100 mm dishes overnight. Cells were harvested after CDDP treatment and washed with PBS. TRIzol^TM^ reagent (Thermo Scientific, 15596026) was used to extract total RNA from each sample. Low-quality sequencing reads and adapters were removed by Cutadapt (v4.4),^71^ and the clean reads were mapped to the human hg38 genome by HISAT2 (v2.2.1)^72^ with default parameters. The gene counts were calculated with gene annotations from the UCSC database using FeatureCounts (v2.0.1).^73^ Fragments Per Kilobase of transcript per Million (FPKM) was introduced to normalize gene expression value. Differential expression analyses were performed with the R DESeq2 package (v1.24.0),^74^ the threshold of *P*-value was 0.05, with fold change ≥1.5. Heatmaps of differentially expressed genes were visualized by R pheatmap package (v1.0.12). GO enrichment analysis was conducted using DAVID (v6.8)^75^ with the default parameters, and visualized by R ggplot2 package (v3.3.5).

### Real-time quantitative PCR (RT-qPCR)

HCT-116 and Hep G2 cells stably transfected with sh*SCR* or sh*TFRC* were seeded in 6-well plates overnight. After CDDP treatment, total RNA from each sample was extracted using TRIzol^TM^ reagent and cDNA was prepared with 5×All-In-One-RT MasterMix (Abm, G490). The RT-qPCR was performed using Taq Pro Universal SYBR qPCR Master Mix (Vazyme Biotech, Q712) on QuantStudio^TM^ 7 Flex system (Thermo Scientific, USA). The relative mRNA expression level was recorded and analyzed with QuantStudio^TM^ Real-Time PCR software by the 2^−^^ΔΔCT^ method.

The primer pairs were used as follows:

*XPC* forward: 5’-CTTCGGAGGGCGATGAAAC-3’;

*XPC* reverse: 5’-TTGAGAGGTAGTAGGTGTCCAC-3’;

*BBC3* forward: 5’-GACCTCAACGCACAGTACGAG-3’;

*BBC3* reverse: 5’-AGGAGTCCCATGATGAGATTGT-3’;

*CDKN1A* forward: 5’-TGTCCGTCAGAACCCATGC-3’;

*CDKN1A* reverse: 5’-AAAGTCGAAGTTCCATCGCTC-3’;

*DDB2* forward: 5’-ACCTCCGAGATTGTATTACGCC-3’

*DDB2* reverse: 5’-TCACATCTTCTGCTAGGACCG-3’

*ERCC5* forward: 5’-CACCAAGCGCAGAAGAACATT-3’;

*ERCC5* reverse: 5’-ACCACTCTCCTTGACTCTACCT-3’;

*NUFIP2* forward: 5’-GGTGAACTAAACGGTAATGCTGG-3’;

*NUFIP2* reverse: 5’-GCTAGTGTCTACAACTTGCTGG-3’;

*ACTIN* forward: 5’-CTCGCCTTTGCCGATCC-3’;

*ACTIN* reverse: 5’-ATCCTTCTGACCCATGCCC-3’.

### Dual-luciferase reporter assay

HCT-116 cells following stable transfection with sh*SCR* or sh*TFRC* were seeded in 12-well plates and transiently transfected with a pGL3-Basic reporter plasmid containing *XPC* promoter together with the Renilla luciferase (pRL-CMV) plasmid using Fugene HD transfection reagent for 24 h. Subsequent to being treated with CDDP, Dual Luciferase Reporter Assay Kit (Vazyme Biotech, DL101) was used for determination of luciferase activity, detecting with a microplate spectrophotometer (SpectraMax M4, Molecular Devices, USA). Firefly luciferase values were normalized with renilla luciferase values.

### RNA-FISH analysis

RNA-FISH analysis was performed for paraffin sections from HCT-116 xenografted tumors stably transfected with sh*SCR* or sh*TFRC* using a customized FISH kit (Boster, MK3965, specifically produced for detecting *XPC*). In brief, the slides were deparaffinized and hydrated, followed by pepsin digestion for 30 min at 37 °C. After that, the slides were post-fixed with 4% paraformaldehyde for 5 min, incubated with pre-hybridization buffer for 3 h at 42 °C and hybridization buffer (containing probes targeting *XPC* mRNA) overnight at 42 °C, and then washed sequentially with 2×, 1×, and 0.2× saline-sodium citrate buffers. After sequential incubation with blocking buffer, biotinylated digoxin, streptavidin-biotin complex-FITC and 4 times washes with PBS, the slides were sealed with antifading mounting medium containing DAPI. Images were captured using a laser scanning confocal microscope (ZIESS-LSM700, German). At least 5 fields in the slides of each group were photographed. RNA-FISH signal was quantified using ImageJ (v1.52).

The sequences of the *XPC* mRNA targeting probes were used as follows:

(1) 5’-TCTATCGAAATAACATCTGCAGCCAGCCAGATCTGCATGC-3’

(2) 5’-GCTTTACCAGAGTGCTGCCTCGAGATGTGGACACCTACTA-3’

(3) 5’-TAGACCAGTGGCTAGAGGTGTTCTGTGAGCAGGAGGAAAA-3’

### Statistical analysis

All quantitative data are presented as the mean ± SEM. Unpaired two-tailed Student’s *t*-test was used to evaluate statistical differences for two groups with one variable. One-way ANOVA analysis with Tukey multiple-comparison test was used to evaluate statistical differences for more than two groups with one variable. Two-way ANOVA analysis with Tukey multiple-comparison test was used to evaluate statistical differences for more than two groups with two or more than two variables. Chi-square and Fisher’s exact test were used to evaluate statistical differences for categorical variables. *P*-value less than 0.05 was considered significant. Asterisks denote statistical significance. *P < 0.05; **P < 0.01; ***P < 0.001; and ****P < 0.0001.

## Supplementary information

Supplementary Materials

Uncropped images of Western Blots and Gels

## Data Availability

The CUT&Tag data and the RNA-seq data have been deposited in the NCBI Gene Expression Omnibus (GSE271155). The mass spectrometry proteomics data have been deposited in the ProteomeXchange Consortium via the PRIDE^76^ partner repository with the dataset identifier PXD063330. Other data are available from the supplementary material or from the corresponding authors upon request.
