# Supplementary material for: Transferrin receptor 1 nuclear translocation facilitates tumor progression via p53-mediated chromatin interactions and genome-wide alterations
Source: Signal Transduct Target Ther. 2025 Jul 8;10:212. doi: 10.1038/s41392-025-02297-6 (PMC12234710; doi:10.1038/s41392-025-02297-6)
Supplement: Supplementary file 1 — Supplementary Materials [file 41392_2025_2297_MOESM1_ESM.docx]

Supplementary Materials for

**Transferrin receptor 1 nuclear translocation facilitates tumor progression via p53-mediated chromatin interactions and genome-wide alterations**

Yaxin Hou^1,#^, Guoheng Tang^1,2,#^, Qizhi Wang^2,3^, Meng Zhou^1^, Ran Xu^1,2^, Xuehui Chen^1^, Guizhi Shi^4^, Zhuoran Wang^1^, Xiyun Yan^1,2,5^*, Jie Zhuang^6^* & Kelong Fan^1,2,5^*

Correspondence to: Kelong Fan, Email: fankelong@ibp.ac.cn; Jie Zhuang, Email: zhuangj@nankai.edu.cn Xiyun Yan, Email: yanxy@ibp.ac.cn.

**This PDF file includes the following:**

Figures S1 to S25 and Supplementary Table S1

Figure. S1.


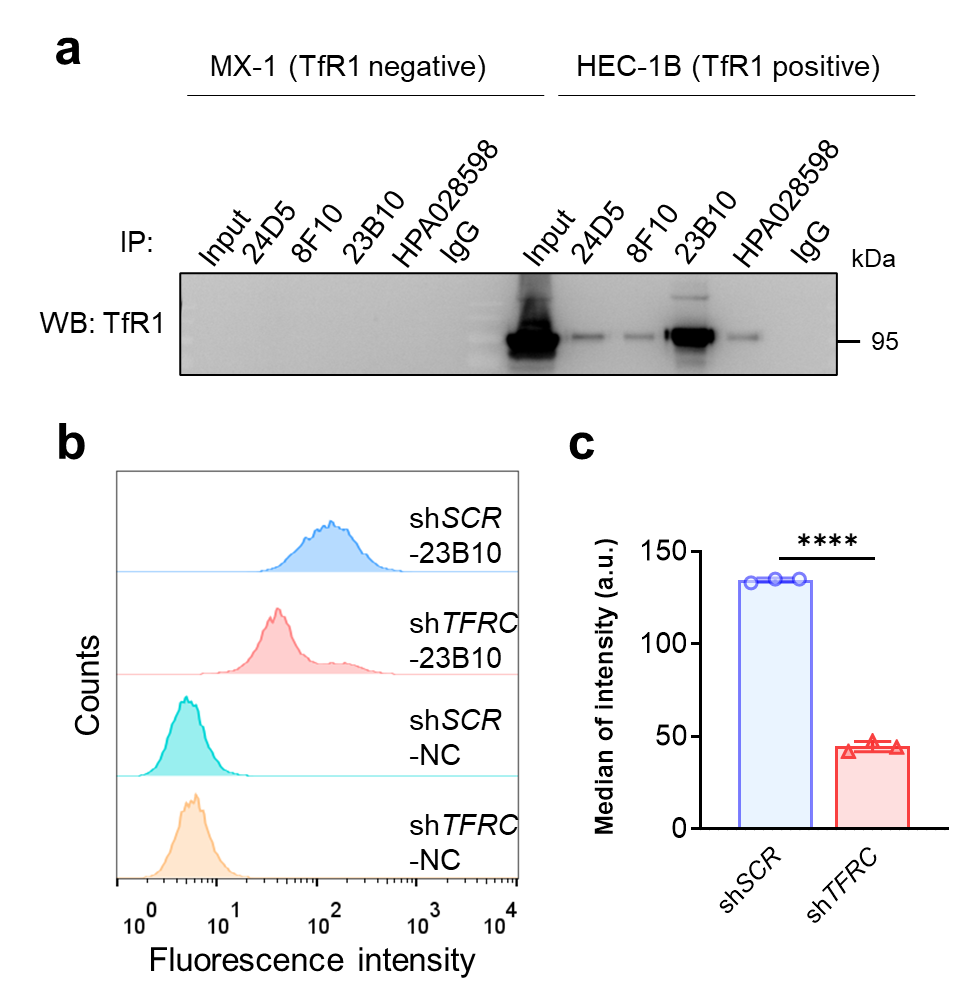


**Supplementary Figure 1. 23B10 anti-TfR1 antibody shows specificity in recognizing human TfR1.** **a** Characterization of 23B10 antibody used for Co-IP in MX-1 (TfR1 negative) cells and HEC-1B (TfR1 positive) cells. The cell lysates were immunoprecipitated with 23B10 antibody, other clones (24D5 and 8F10) produced from the same batch, and a commercial anti-TfR1 antibody (Sigma, HPA028598). **b** Flow cytometry histogram of HCT-116 sh*SCR* and sh*TFRC* cells incubated with 23B10 antibody and fluorescent secondary antibody or incubated with fluorescent secondary antibody only (NC). **c** Quantification of the median fluorescence intensity in (**b**) (*n* = 3). *P*-value was determined using unpaired two-tailed Student’s t-test. *****P* < 0.0001.

Figure. S2.

**Supplementary Figure 2. Nuclear TfR1 expression is detected in the majority of tumor tissues.** Statistical diagram of nuclear stating of TfR1 in IHC analysis on multi-organ cancer tissue microarray (*n* = 204).

Figure. S3.

**
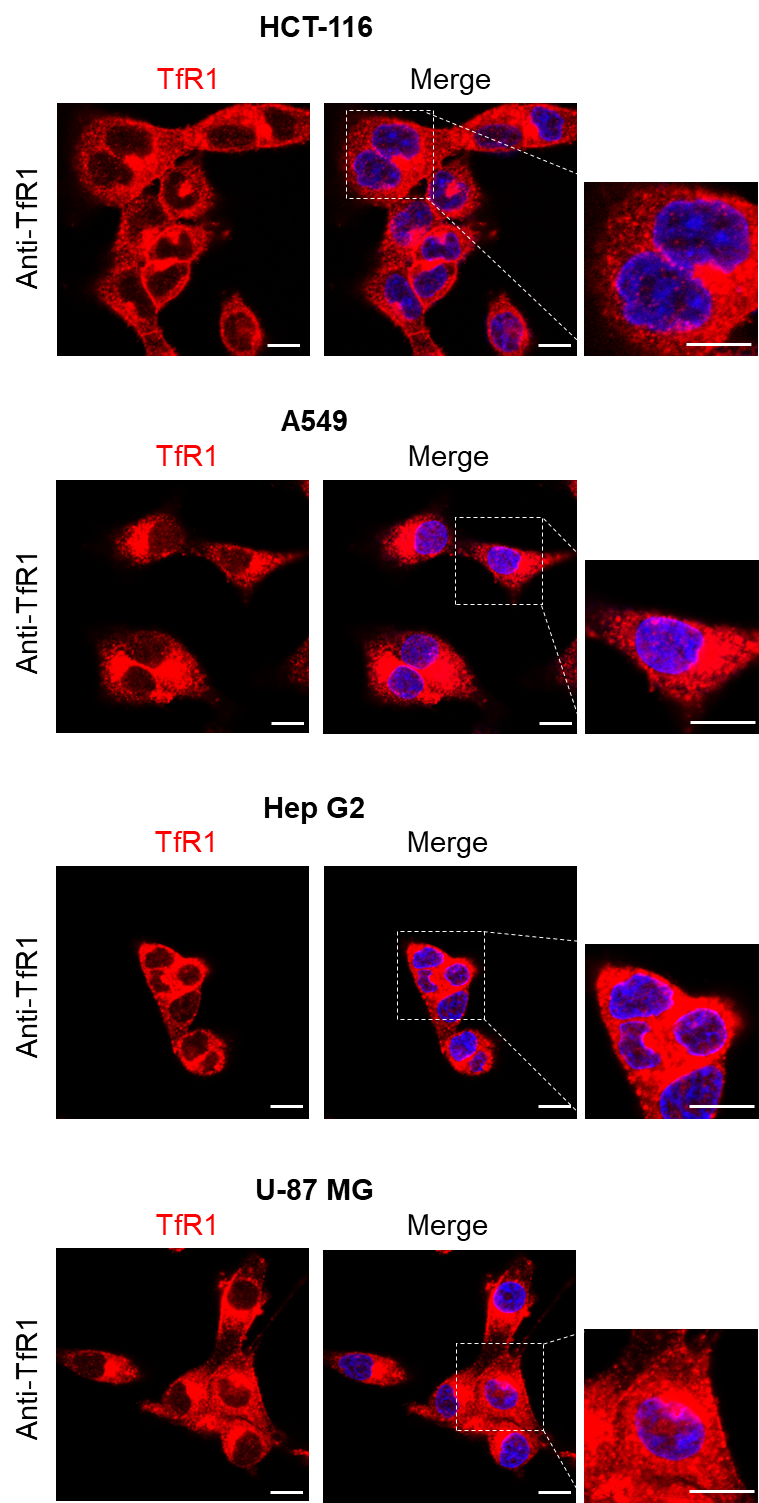
**

**Supplementary Figure 3. TfR1 is localized in the nucleus in various tumor cell lines.** Confocal microscopy images of TfR1 in HCT-116 cells, A549 cells, Hep G2 cells, and U-87 MG cells stained with anti-TfR1 antibody. Nuclei were stained with DAPI. Scale bar = 10 μm.

Figure. S4.


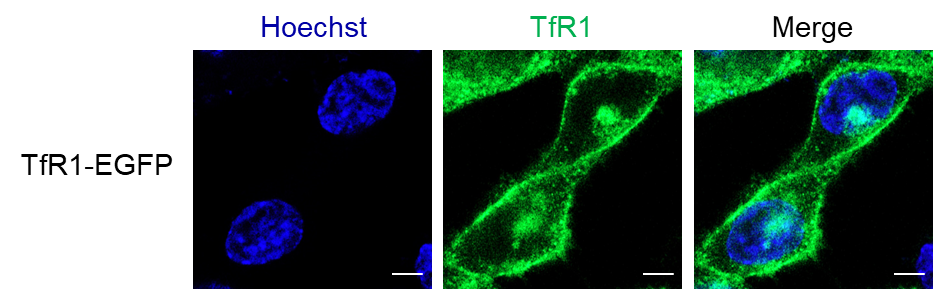


**Supplementary Figure 4. Exogenous transfected TfR1 localizes in the nucleus.** Confocal microscopy images of TfR1-EGFP expression HCT-116 cells. Nuclei were stained with Hoechst. Scale bar = 5 μm.

Figure. S5.


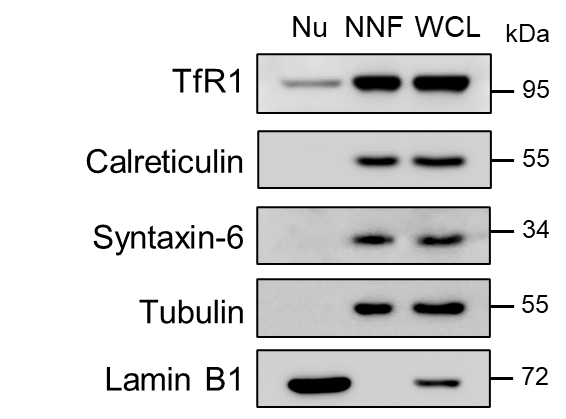


**Supplementary Figure 5. TfR1 in the nucleus is not contaminated by the ER and the Golgi complex components.** Immunoblot analysis of subcellular fractionation of HCT-116 cells detecting the ER component (Calreticulin) and the Golgi complex component (Syntaxin-6) in the nuclear fraction. Lamin B1 was used as Nu loading control. Tubulin was used as NNF and WCL loading control.

Figure. S6.


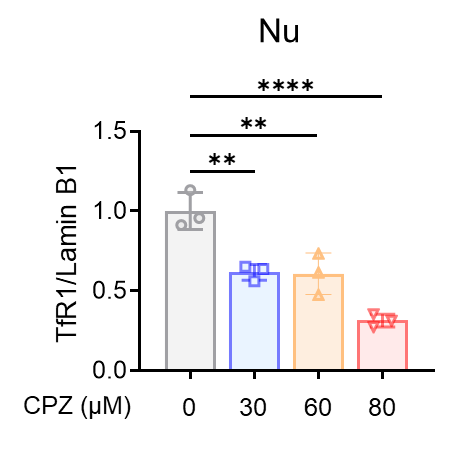


**Supplementary Figure 6. Quantification of the ratio of TfR1 to Lamin B1 in the nucleus after CPZ treatment in Fig. 1d (*n* = 3).** *P*-value was determined using one-way ANOVA analysis. ***P* < 0.01; *****P* < 0.0001.

Figure. S7.


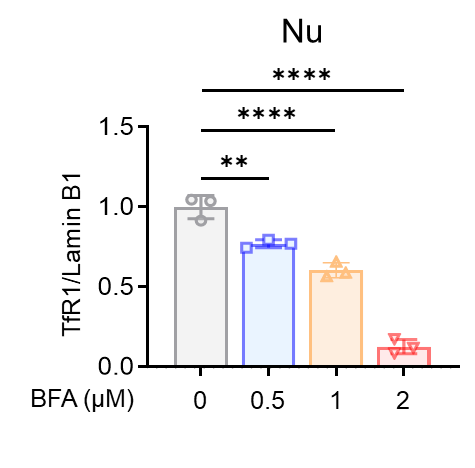


**Supplementary Figure 7. Quantification of the ratio of TfR1 in the nucleus after BFA to Lamin B1 treatment in Fig. 1e (*n* = 3).** *P*-value was determined using one-way ANOVA analysis. ***P* < 0.01; *****P* < 0.0001.

Figure. S8.

*
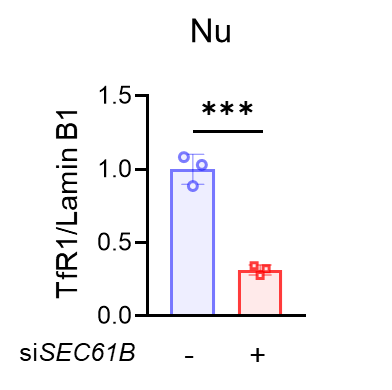
*

**Supplementary Figure 8. Quantification of the ratio of TfR1 to Lamin B1 in the nucleus upon Sec61β knockdown** **in Fig. 1g (*n* = 3).** *P*-value was determined using unpaired two-tailed Student’s t-test. ****P* < 0.001.

Figure. S9.


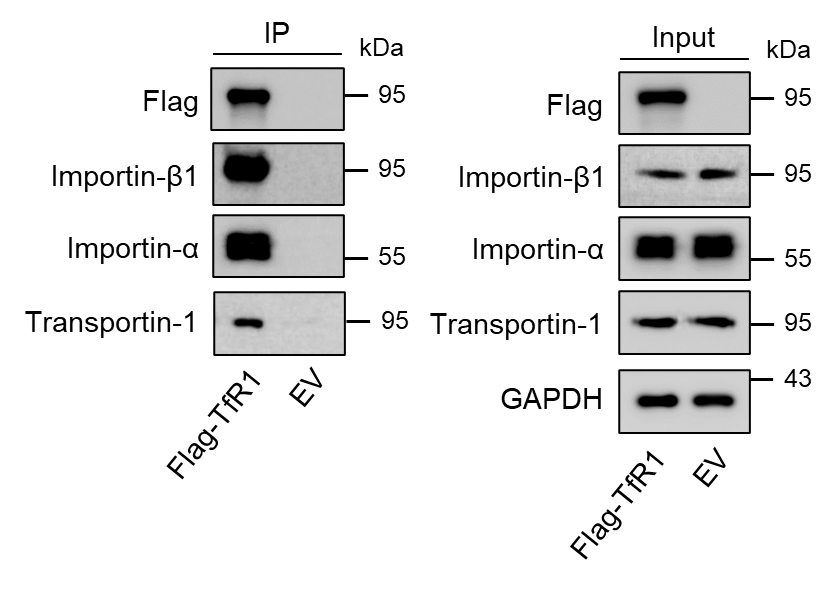


**Supplementary Figure 9. TfR1 interacts with several classical nuclear transporters.** Co-IP analysis of the interactions between Flag-TfR1 and importin-β, importin-α or transportin-1. GAPDH was used as loading control.

Figure. S10.


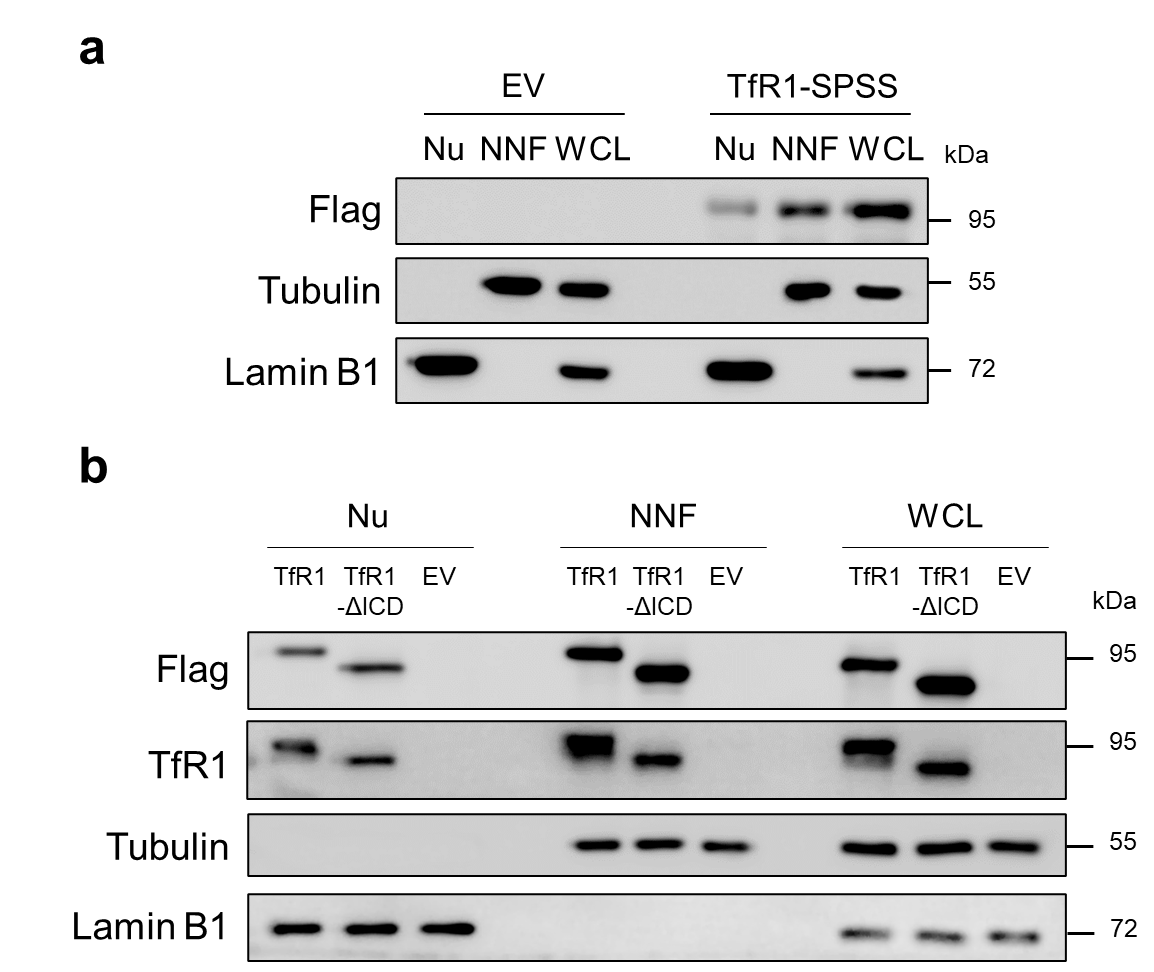


**Supplementary Figure 10. The intracellular domain of TfR1 is not essential for its nuclear translocation. a** Immunoblot analysis of nuclear and cytoplasmic fractions of HCT-116 cells transfected with TfR1-SPSS plasmids (mutating residues 58-61 from KPKR to SPSS). **b** Subcellular fractionation of MX-1 cells transfected with TfR1-ΔICD (deleting residues 1-67) or full-length TfR1 plasmids. Lamin B1 was used as Nu loading control. Tubulin was used as NNF and WCL loading control.

Figure. S11.


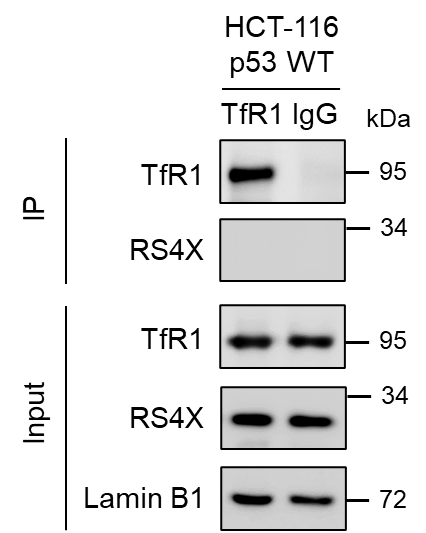


**Supplementary Figure 11. TfR1 does not interact with ribosomal protein S4X (RS4X) in the nucleus.** Co-IP analysis of the interaction between TfR1 and RS4X in HCT-116 cells. Lamin B1 was used as loading control.

Figure. S12.


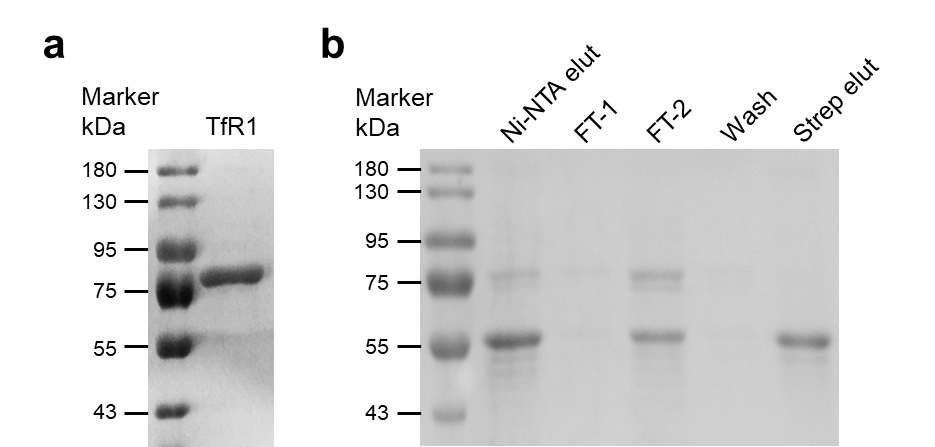


**Supplementary Figure 12. Characterization of recombinant human TfR1 and p53 purifications. a** SDS-PAGE analysis of recombinant ectodomain of human TfR1 purified by size-exclusion chromatography. **b** SDS-PAGE analysis of recombinant human p53 sequentially purified by Ni-NTA affinity chromatography and strep-tactin affinity chromatography. Ni-NTA elut: Elution fraction from Ni-NTA affinity column; FT: Flow through fraction; Strep elut: Elution fraction from strep-tactin affinity column.

Figure. S13.


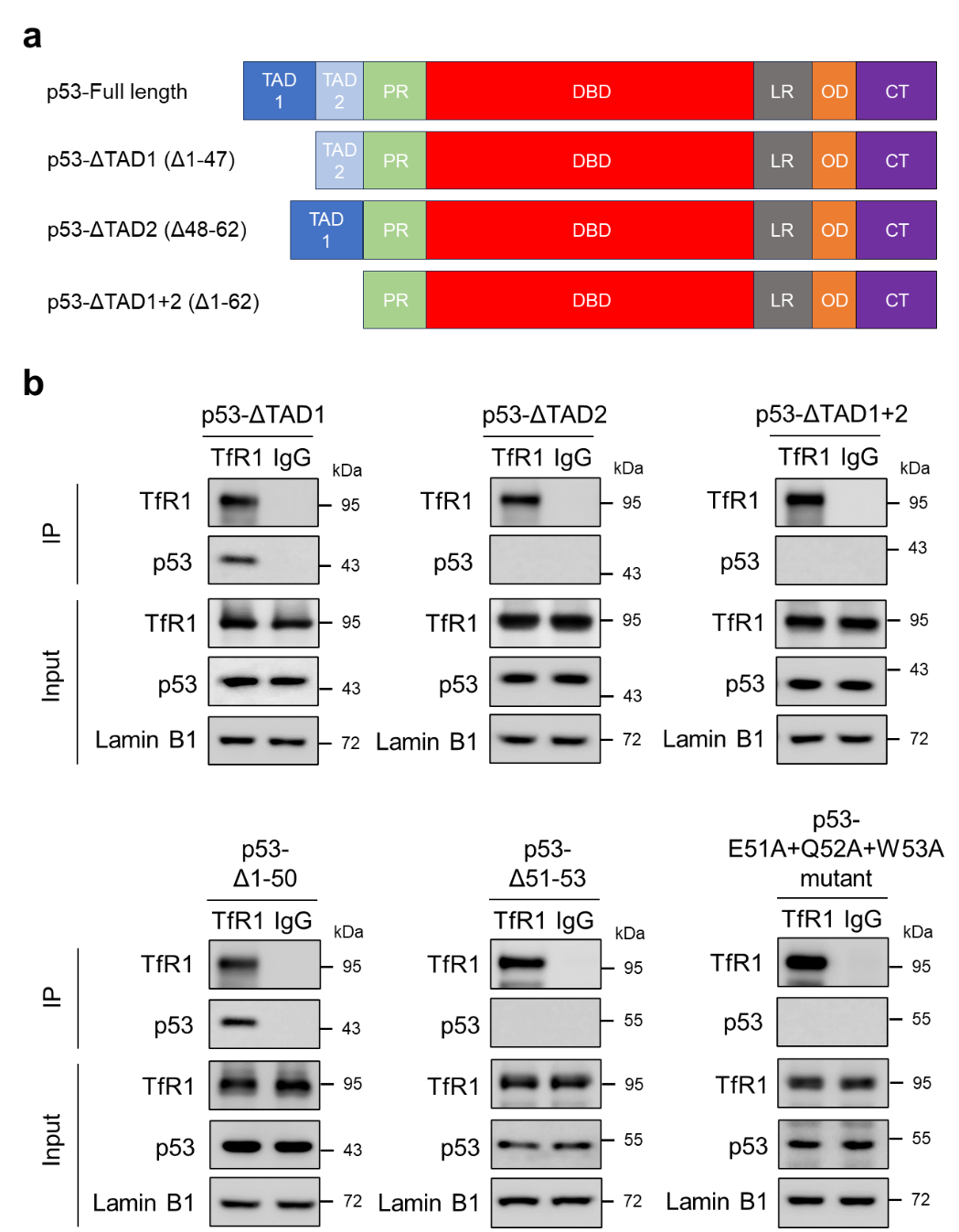


**Supplementary Figure 13. Determination of the p53 binding region responsible for the interaction with TfR1.** **a** Schematic illustration of p53 truncation constructs: p53-ΔTAD1, p53-ΔTAD2, and p53-ΔTAD1+2. **b** Co-IP analysis of the interaction between TfR1 and various p53 truncations or mutants. Lamin B1 was used as loading control.

Figure. S14.


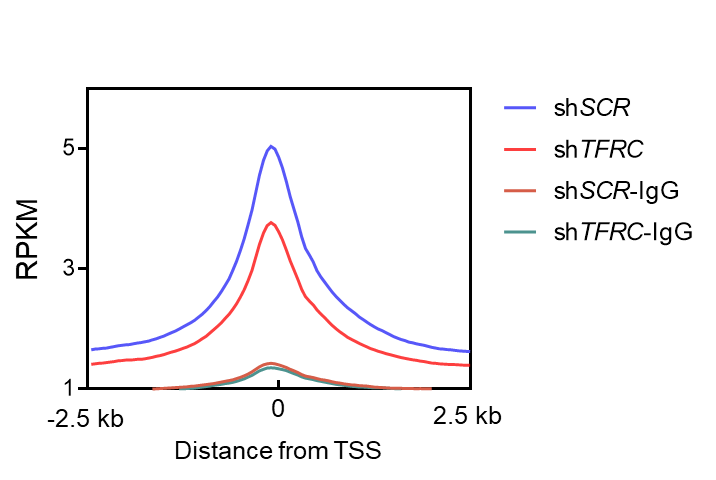


**Supplementary Figure 14. Comparison of CUT&Tag read density plots** **at TfR1-bound gene loci between HCT-116 sh*SCR* and sh*TFRC* cells.** The data were from the same batch of experiments as Fig. 3h, therefore using the same sh*SCR* group as the control.

Figure. S15.


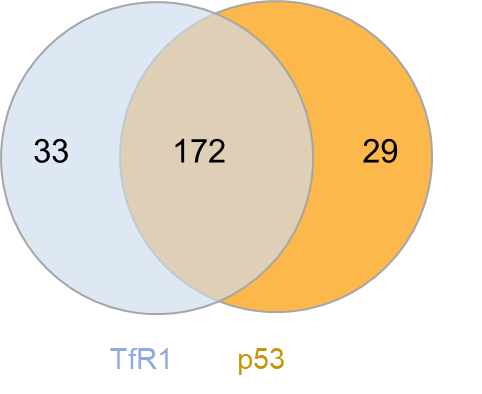


**Supplementary Figure 15. Nuclear TfR1 and p53 exhibit high similarity in the binding of known motifs.** Venn diagram overlap analysis of known motifs between TfR1 and p53.

Figure. S16.


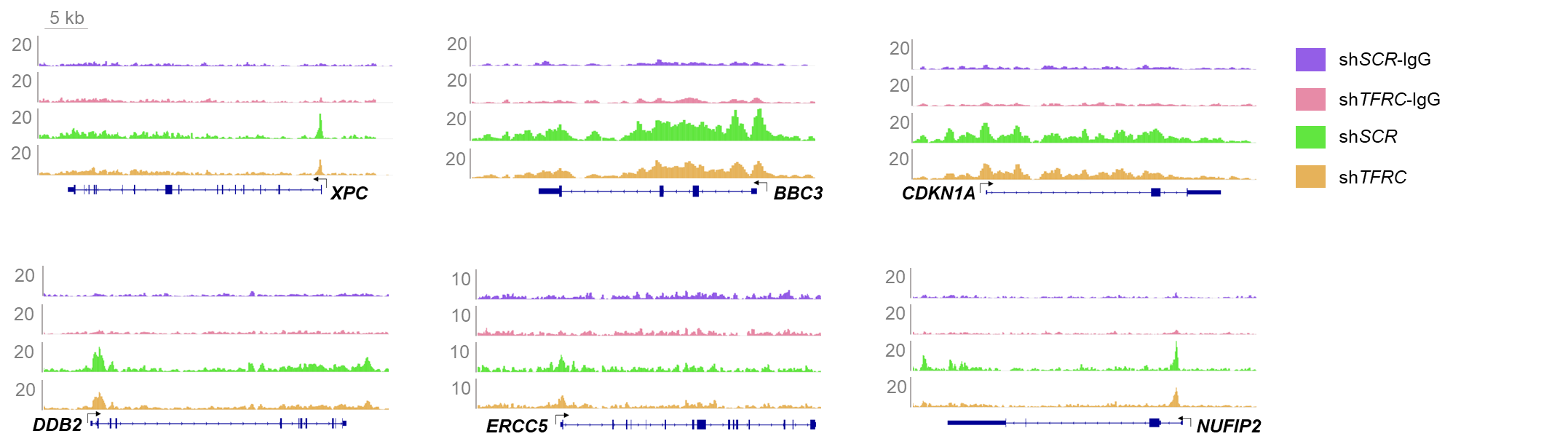


**Supplementary Figure 16. CUT&Tag peak distribution for TfR1 at the** **representative gene loci in HCT-116 sh*SCR* and sh*TFRC* cells.**

Figure. S17.


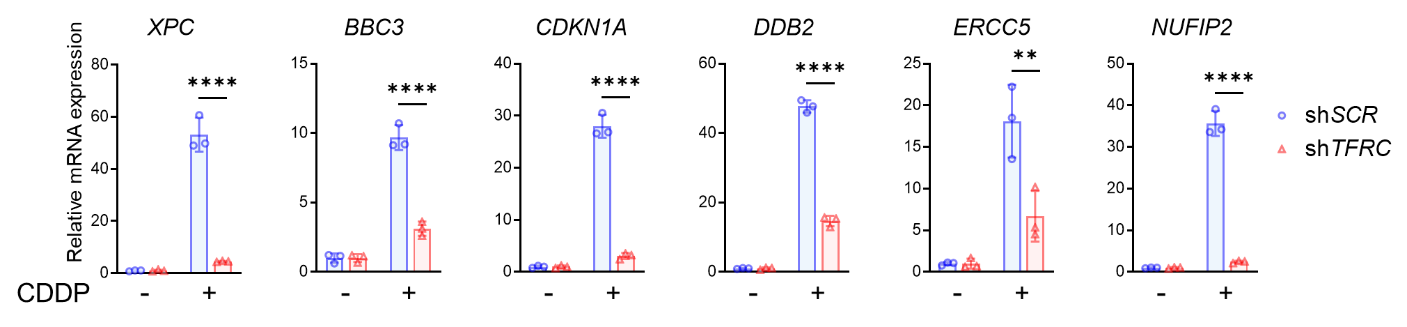


**Supplementary Figure 17. qPCR analysis of the representative DEGs after 25 μM CDDP treatment for 24 h in Hep G2 cells.** *ACTIN* gene was used as an internal control (*n* = 3). *P*-value was determined using two-way ANOVA analysis. ***P* < 0.01; *****P* < 0.0001.

Figure. S18.

**
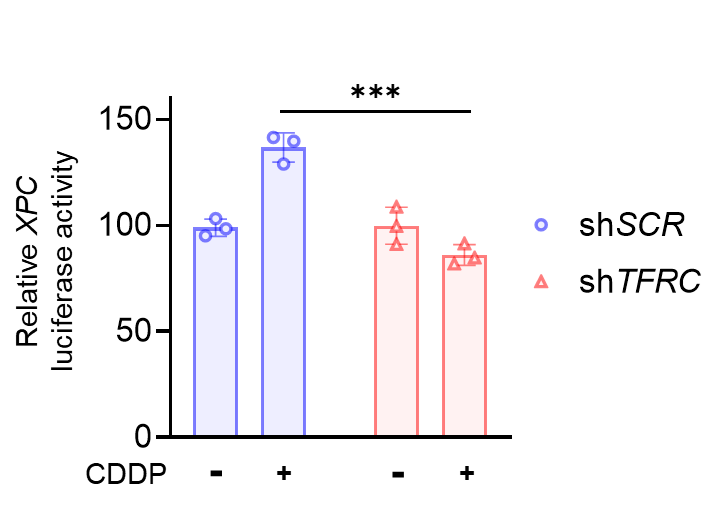
**

**Supplementary Figure 18. TfR1 transcriptionally regulates *XPC* expression upon CDDP stimulation.** HCT-116 cells were transfected with sh*SCR* or sh*TFRC* with or without 50 μM CDDP treatment for 24 h. Luciferase reporter activity was normalized by Renilla luciferase activity in each group (*n* = 3). Data were presented as the mean ± SEM from at least three independent experiments. *P*-value was determined using two-way ANOVA analysis. ****P* < 0.001.

Figure. S19.

*
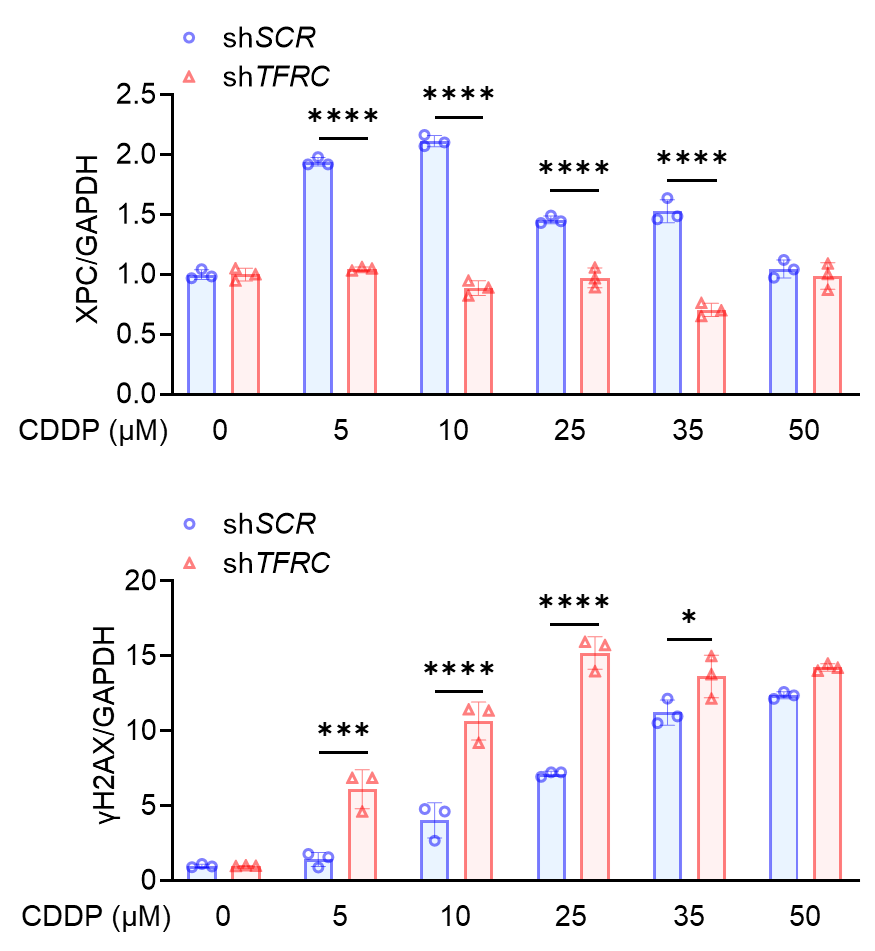
*

**Supplementary Figure 19. Quantification of the alternation rate of XPC and γH2AX to GAPDH in response to CDDP stimulation in HCT-116 sh*SCR* and sh*TFRC* cells in Fig. 4e (*n* = 3).** *P*-value was determined using two-way ANOVA analysis. **P* < 0.05; ****P* < 0.001; *****P* < 0.0001.

Figure. S20.


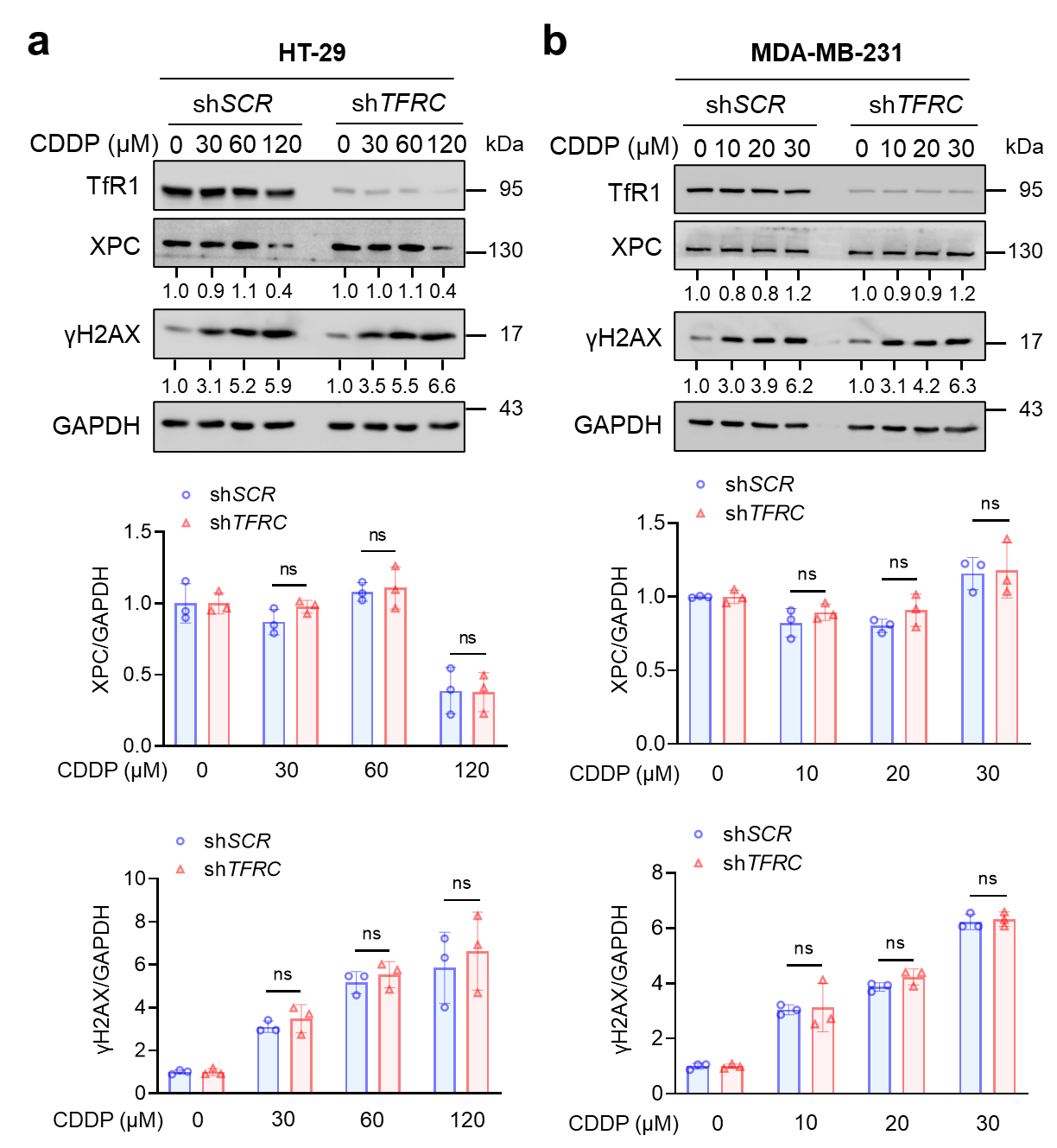


**Supplementary Figure 20. CDDP is incapable of inducing XPC expression in tumor cells with LOF p53 mutations.** **a**, **b** Immunoblot analysis TfR1, XPC, and γH2AX after treated with different concentrations of CDDP treatment for 24 h in HT-29 cells transfected with sh*SCR* or sh*TFRC* (**a**), and in MDA-MB-231 cells transfected with sh*SCR* or sh*TFRC* (**b**). Quantification analysis of XPC and γH2AX in response to CDDP stimulation is shown below (*n* = 3). GAPDH was used as an internal control. The relative abundance of XPC and γH2AX to GAPDH was normalized to the CDDP = 0 controls in sh*SCR* or sh*TFRC* cells. *P*-value was determined using two-way ANOVA analysis. ns, not significant.

Figure. S21.


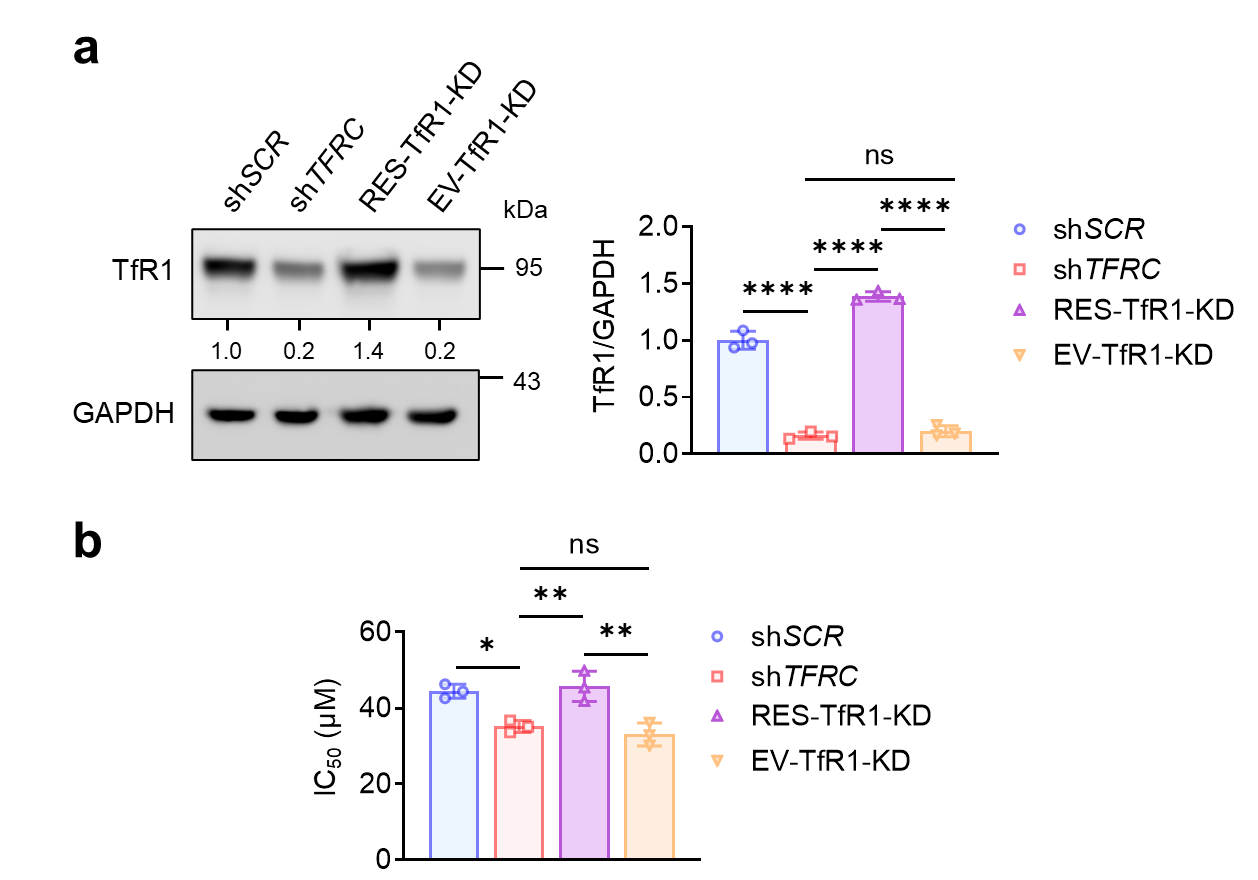


**Supplementary Figure 21. Knockdown of TfR1 in tumor cells expressing functional p53 leads to decreased tolerance to CDDP and can be rescued by TfR1 re-expression.** **a** Immunoblot analysis and quantification analysis of TfR1 in HCT-116 cells transfected with the indicated constructs (*n* = 3). GAPDH was used as an internal control. The relative abundance of TfR1 to GAPDH was normalized to the sh*SCR* cells. **b** Quantification of IC_50_ value after CDDP treatment for 24 h in HCT-116 cells transfected with the indicated constructs (*n* = 3). Data were presented as the mean ± SEM from at least three independent experiments. *P*-value was determined using one-way ANOVA analysis. **P* < 0.05; ***P* < 0.01; *****P* < 0.0001; ns, not significant.

Figure. S22.


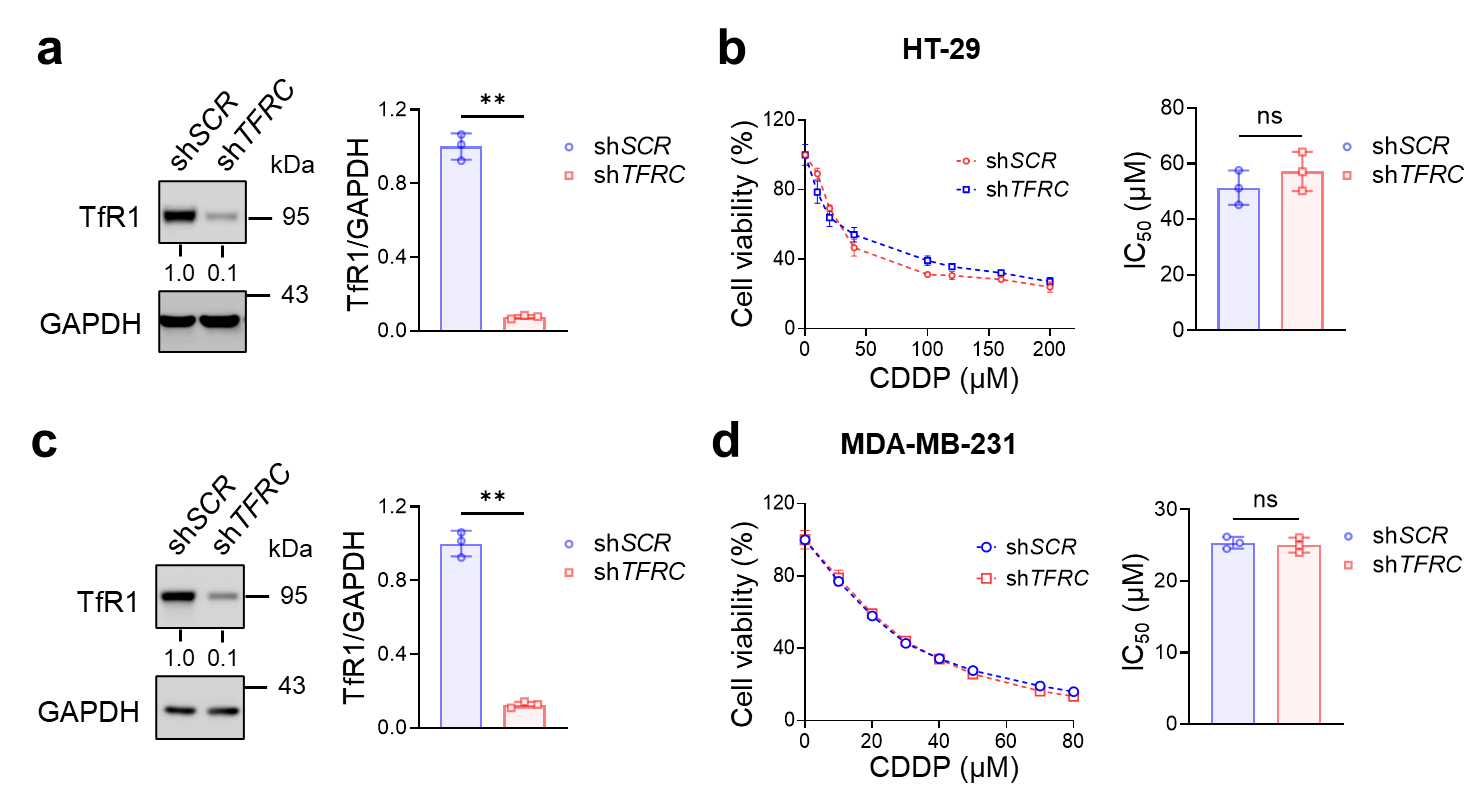


**Supplementary Figure 22. Knockdown of TfR1 in tumor cells with LOF p53 mutations has no effect on tolerance to CDDP. a**, **b** Immunoblot analysis and quantification analysis of TfR1 in (**a**) and cell viability with quantification of IC_50_ value in (**b**) in HT-29 sh*SCR* or sh*TFRC* cells (*n* = 3). **c**, **d** Immunoblot analysis and quantification analysis of TfR1 in (**c**) and cell viability after CDDP treatment for 24 h with quantification of IC_50_ value in (**d**) in MDA-MB-231 sh*SCR* or sh*TFRC* cells (*n* = 3). GAPDH was used as an internal control. The relative abundance of TfR1 to GAPDH was normalized to the sh*SCR* cells. Data were presented as the mean ± SEM from at least three independent experiments. *P*-value was determined using unpaired two-tailed Student’s t-test. ***P*< 0.01; ns, not significant*.*

Figure. S23.


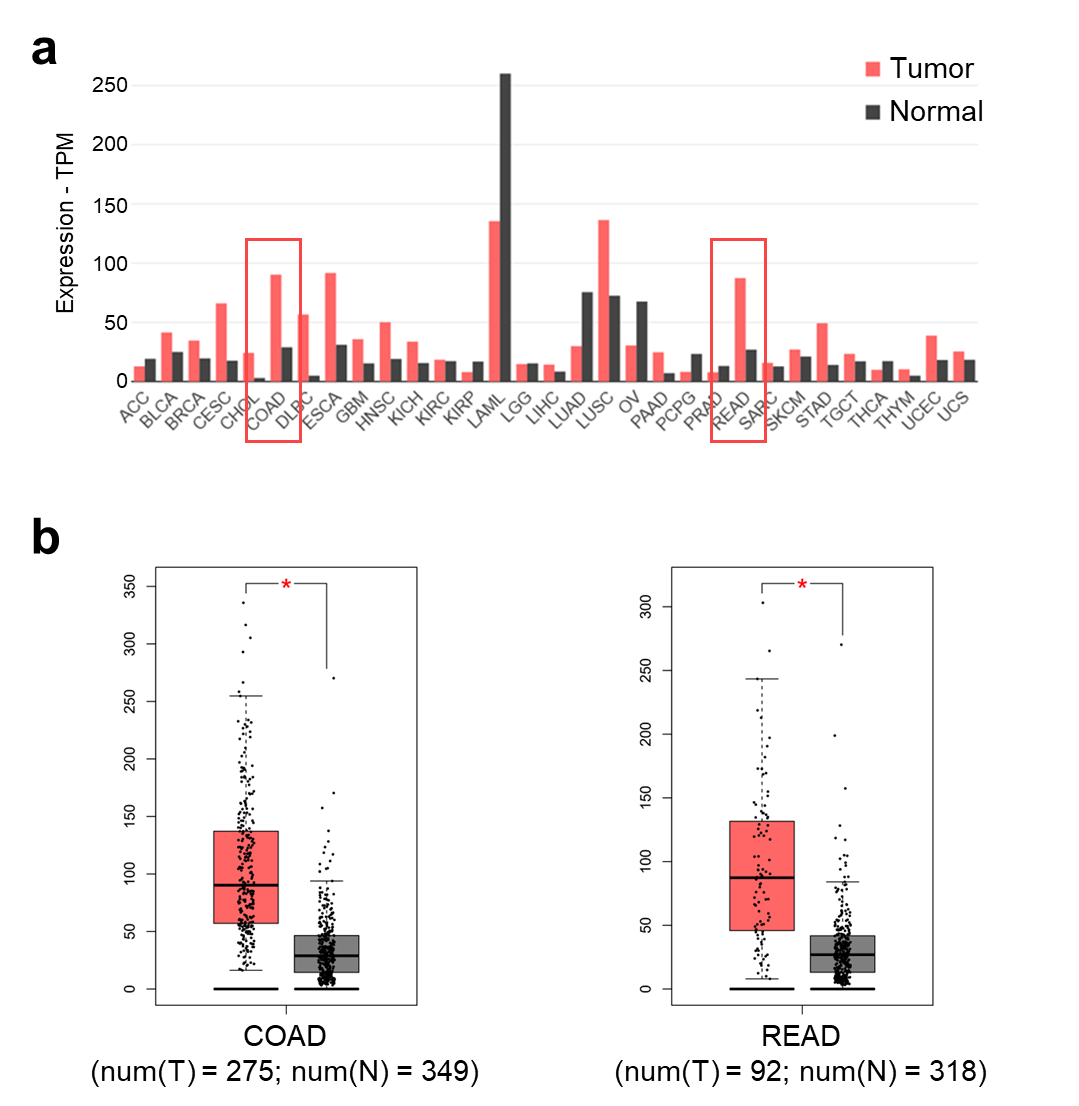


**Supplementary Figure 23. Colon adenocarcinoma and rectum adenocarcinoma exhibit significant differences in TfR1 expression between tumor tissue and normal tissue.** **a** Gene expression profile across all tumor samples and paired normal tissues from the GEPIA database. **b** Gene expression profile of COAD and READ on box plots from the GEPIA database.

Figure. S24.


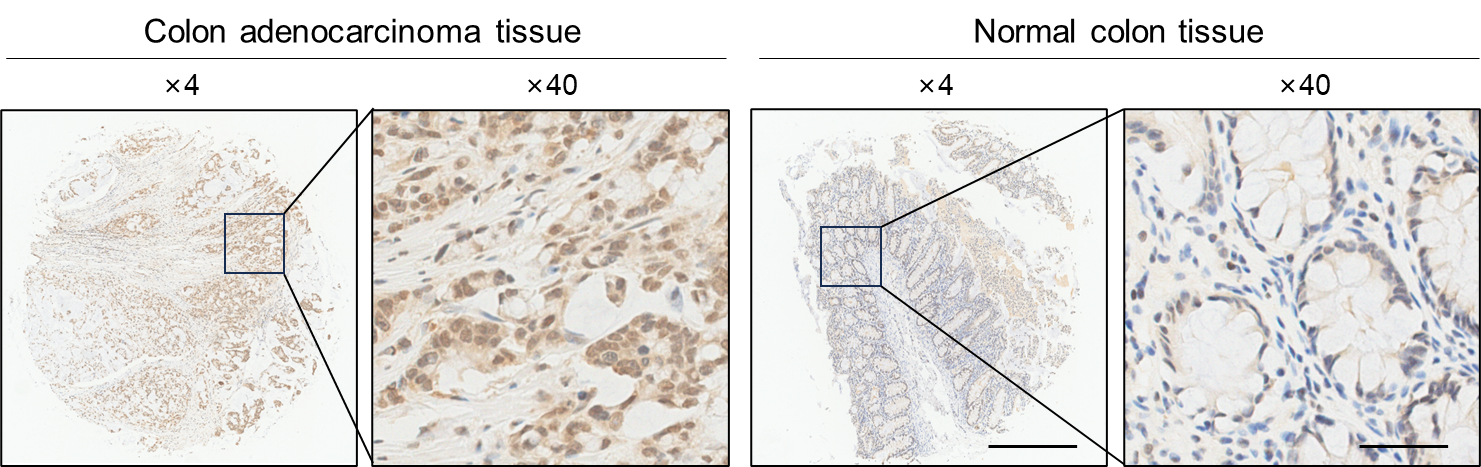


**Supplementary Figure 24. Representative IHC images of TfR1 staining in colon adenocarcinoma tissues and normal colon tissues with low (×4) and high (×40) magnification.** Scale bar = 500 μm (×4); Scale bar = 50 μm (×40).

Figure. S25.


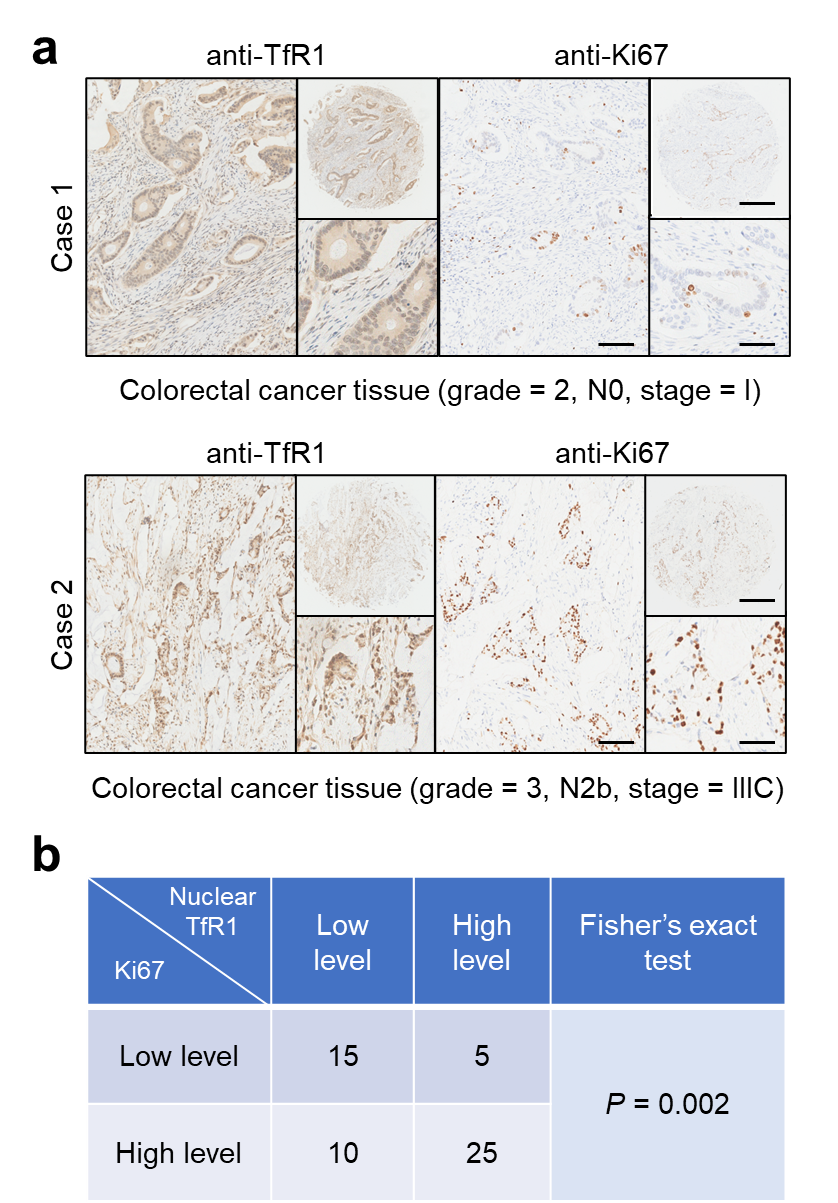


**Supplementary Figure 25. Nuclear TfR1 level is positively associated with Ki-67 level.** **a** Representative 2 cases of IHC images (×2, ×10, and ×20 magnifications) of TfR1 and Ki-67 staining in the CRC tissue microarray. Scale bar = 500 μm (magnification ×2); scale bar = 100 μm (magnification ×10); scale bar = 50 μm (magnification ×20). **b** Chi-square test of the correlation between the levels of nuclear TfR1 and Ki-67 of the IHC staining.

**Supplementary Table 1:** Patient information of tissue microarray.

| **Microarray ID** | **Position** | **Age** | **Gender** | **Histology** | **WHO grades** | **AJCC grades** | **TNM** | **Tumor size (cm)** |
| --- | --- | --- | --- | --- | --- | --- | --- | --- |
| D1060401 | A1 | 48 | female | villoglandular adenocarcinoma | 1 | I | T1N0M0 | - |
|  | A2 | 37 | male | tubular villoglandular adenocarcinoma | 1 | I | T1N0M0 | 4.5×3.0×0.5 |
|  | A3 | 53 | male | adenocarcinoma | 1-2 | I | T2N0N0 | 7×4×4 |
|  | A4 | 52 | male | adenocarcinoma | 2 | I | T2N0M0 | - |
|  | A5 | 64 | male | adenocarcinoma | 2 | I | T2N0M0 | 4.0×3.0×0.5 |
|  | A6 | 42 | male | adenocarcinoma | 2 | I | T2N0N0 | - |
|  | A7 | 61 | male | adenocarcinoma | 2 | I | T2N0M0 | 4.5×4.0×0.5 |
|  | A8 | 55 | female | adenocarcinoma | 2 | I | T2N0M0 | 5.5×4×0.5 |
|  | A9 | 68 | male | adenocarcinoma | 2 | I | T2N0N0 | - |
|  | A10 | 61 | male | adenocarcinoma | 3 | I | T2N0M0 | 4.0×3.5×0.5 |
|  | A11 | 43 | female | adenocarcinoma | 2 | I | T2N0M0 | - |
|  | A12 | 53 | female | tubular villoglandular adenocarcinoma | 1 | I | T2N0M0 | 3.0×2.5×0.5 |
|  | B1 | 67 | male | mucinous adenocarcinoma | 2 | I | T2N0M0 | 4.0×3.5×0.6 |
|  | B2 | 51 | male | adenocarcinoma | 2 | I | T2N0M0 | 4.0×4.0×1.0 |
|  | B3 | 61 | female | adenocarcinoma | 2 | I | T2N0M0 | 4.5×3.5×1.1 |
|  | B4 | 50 | male | adenocarcinoma | 2 | IIA | T3N0M0 | 4×2.5×1 |
|  | B5 | 64 | male | adenocarcinoma | 2 | IIA | T3N0M0 | - |
|  | B6 | 75 | female | adenocarcinoma | 3 | IIA | T3N0M0 | 5×5×0.5 |
|  | B7 | 51 | male | adenocarcinoma | 2 | IIA | T3N0M0 | 5×5×0.8 |
|  | B8 | 42 | female | adenocarcinoma | 2 | IIA | T3N0M0 | - |
|  | B9 | 70 | male | adenocarcinoma | 3 | IIA | T3N0M0 | 5.0×4.5×1.0 |
|  | B10 | 53 | male | villoglandular adenocarcinoma | 1 | IIA | T3N0M0 | - |
|  | B11 | 68 | male | tubular villoglandular adenocarcinoma | 1 | IIA | T3N0M0 | 6.0×4.5×0.5 |
|  | B12 | 34 | male | adenocarcinoma | 2 | IIA | T3N0M0 | 4.5×3.5×0.5 |
|  | C1 | 59 | female | adenocarcinoma | 2 | IIA | T3N0M0 | 4.5×4.0×1.0 |
|  | C2 | 37 | male | adenocarcinoma | 2 | IIA | T3N0M0 | 6×6×3.5 |
|  | C3 | 57 | female | adenocarcinoma | 2 | IIA | T3N0M0 | - |
|  | C5 | 62 | female | villoglandular adenocarcinoma | 1 | IIA | T3N0M0 | 5×5×3 |
|  | C6 | 69 | male | adenocarcinoma | 3 | IIA | T3N0M0 | 3.5×3.5×0.5 |
|  | C7 | 60 | male | adenocarcinoma | 2 | IIA | T3N0M0 | 4.0×4.0×0.5 |
|  | C8 | 55 | male | adenocarcinoma | 3 | IIA | T3N0M0 | 3.0×2.5×1.1 |
|  | C9 | 65 | female | adenocarcinoma | 3 | IIA | T3N0M0 | 4.5×3.5×0.5 |
|  | C10 | 48 | female | adenocarcinoma | 3 | IIA | T3N0M0 | 5×4.0×1.5 |
|  | C11 | 57 | male | adenocarcinoma | 2 | IIA | T3N0M0 | 3.5×3×1 |
| D1060401 | C12 | 53 | male | villoglandular adenocarcinoma | 1 | IIB | T4aN0M0 | 5×5×1 |
|  | D1 | 48 | male | adenocarcinoma | 2 | IIB | T4aN0M0 | - |
|  | D2 | 52 | female | adenocarcinoma | 3 | IIB | T4aN0M0 | - |
|  | D3 | 75 | male | adenocarcinoma | 2 | IIB | T4aN0M0 | 4×3×0.5 |
|  | D4 | 60 | female | mucinous adenocarcinoma | 3 | IIB | T4aN0M0 | 7×6×1.5 |
|  | D5 | 48 | female | villoglandular adenocarcinoma | 2 | IIB | T4aN0M0 | 5×4.5×1.5 |
|  | D6 | 75 | male | villoglandular adenocarcinoma | 2 | IIB | T4aN0M0 | 10×9×4.5 |
|  | D7 | 51 | male | adenocarcinoma | 1-2 | IIB | T4aN0M0 | 7×5×0.5 |
|  | D8 | 49 | female | adenocarcinoma | 1-2 | IIB | T4aN0M0 | 6×6×5 |
|  | D9 | 63 | male | adenocarcinoma | 3 | IIB | T4aN0M0 | - |
|  | D10 | 53 | female | adenocarcinoma | 2 | IIB | T4aN0M0 | - |
|  | D11 | 56 | male | tubular villoglandular adenocarcinoma | 1-2 | IIB | T4aN0M0 | 4.0×3.5×0.5 |
|  | D12 | 48 | female | adenocarcinoma | 3 | IIIA | T2N1aM0 | - |
|  | E1 | 43 | male | adenocarcinoma | 2 | IIIA | T2N1aM0 | 5×3.8×0.5 |
|  | E2 | 63 | male | mucinous adenocarcinoma | 2 | IIIA | T2N1aM0 | 4.0×4.0×0.5 |
|  | E3 | 40 | male | adenocarcinoma | 2 | IIIA | T2N1aM0 | 3×1.8×0.5 |
|  | E4 | 68 | male | adenocarcinoma | 2 | IIIA | T2N1aM0 | - |
|  | E5 | 48 | female | adenocarcinoma | 2 | IIIB | T3N1aM0 | 6.0×5.0×0.5 |
|  | E6 | 78 | male | mucinous adenocarcinoma | 2 | IIIB | T3N1aM0 | 3.0×3.0×0.5 |
|  | E7 | 44 | male | mucinous adenocarcinoma | 3 | IIIB | T3N1aM0 | 5.0×3.0×0.5 |
|  | E8 | 48 | male | adenocarcinoma | 3 | IIIB | T3N1aM0 | 5.5×4.5×1.4 |
|  | E9 | 60 | male | adenocarcinoma | 2-3 | IIIB | T3N1aM0 | - |
|  | E10 | 78 | male | adenocarcinoma | 2 | IIIB | T3N1aM0 | - |
|  | E11 | 50 | male | adenocarcinoma | 2 | IIIB | T3N1aM0 | - |
|  | E12 | 65 | male | adenocarcinoma | 1-2 | IIIB | T3N1aM0 | - |
|  | F1 | 37 | male | tubular villoglandular adenocarcinoma | 2 | IIIB | T3N1aM0 | - |
|  | F2 | 55 | female | adenocarcinoma | 2 | IIIB | T3N1bM0 | 4.5×4.0×1.0 |
|  | F3 | 66 | male | adenocarcinoma | 2 | IIIB | T3N1bM0 | 4×3×1 |
|  | F4 | 65 | female | adenocarcinoma | 2 | IIIB | T3N1bM0 | 5.5×4.5×0.5 |
|  | F5 | 22 | female | adenocarcinoma | 2 | IIIB | T3N1bM0 | 3×2.7×0.7 |
|  | F6 | 40 | female | adenocarcinoma | 3 | IIIB | T3N1bM0 | 5.5×3.5×1.0 |
|  | F7 | 59 | female | adenocarcinoma | 2 | IIIB | T3N1bM0 | 3.5×3.0×0.5 |
|  | F8 | 54 | male | adenocarcinoma | 2 | IIIB | T3N1bM0 | 10×5.0×2 |
|  | F9 | 43 | male | adenocarcinoma | 1-2 | IIIB | T3N1bM0 | 7.5×4.5×1.3 |
|  | F10 | 65 | female | tubular villoglandular adenocarcinoma | 1 | IIIB | T3N1bM0 | - |
|  | F11 | 60 | male | tubular villoglandular adenocarcinoma | 1-2 | IIIB | T3N1bM0 | - |
|  | F12 | 59 | male | adenocarcinoma | 2 | IIIB | T3N1cM0 | 5.0×3.5×0.6 |
|  | G3 | 60 | female | villoglandular adenocarcinoma | 2 | IIIB | T3N2aM0 | 3.5×3.0×0.5 |
| D1060401 | G4 | 50 | male | adenocarcinoma | 3 | IIIB | T3N2aM0 | 6.5×5.0×3.5 |
|  | G5 | 42 | female | adenocarcinoma | 2 | IIIB | T3N2aM0 | - |
|  | G6 | 33 | female | tubular villoglandular adenocarcinoma | 2 | IIIB | T3N2aM0 | 5×4.5×1 |
|  | G7 | 36 | female | villoglandular adenocarcinoma | 1 | IIIB | T3N2aM0 | 3×2×1.3 |
|  | G8 | 58 | male | villoglandular adenocarcinoma | 1 | IIIB | T3N2aM0 | - |
|  | G9 | 30 | male | mucinous adenocarcinoma | 3 | IIIB | T3N2aM0 | - |
|  | G10 | 53 | female | adenocarcinoma | 2 | IIIB | T3N2aM0 | - |
|  | G11 | 68 | male | mucinous adenocarcinoma | 2-3 | IIIB | T4aN1aM0 | 6.5×6×2.5 |
|  | H1 | 66 | male | adenocarcinoma | 2 | IIIB | T4aN1bM0 | 5×3×2 |
|  | H2 | 52 | female | villoglandular adenocarcinoma | 2 | IIIB | T4aN1cM0 | 6×4×1 |
|  | H3 | 60 | male | adenocarcinoma | 2 | IIIC | T3N2bM0 | 6.5×3.0×0.5 |
|  | H4 | 57 | male | villoglandular adenocarcinoma | 2 | IIIC | T3N2bM0 | 3×3×1.5 |
|  | H5 | 31 | female | adenocarcinoma | 2 | IIIC | T3N2bM0 | 4.5×3.0×3.0 |
|  | H6 | 76 | male | adenocarcinoma | 2 | IIIC | T4aN2aM0 | 3.0×3.0×0.5 |
|  | H7 | 50 | male | adenocarcinoma | 2 | IIIC | T4aN2aM0 | 4×3×1 |
|  | H8 | 74 | male | adenocarcinoma | 2 | IIIC | T4aN2aM0 | 5.5×1.2×2.0 |
|  | H9 | 68 | male | mucinous adenocarcinoma | 2-3 | IIIC | T4bN1cM0 | 5.0×3.5×0.5 |
|  | H10 | 32 | male | adenocarcinoma | 2 | IIIC | T4bN1cM0 | 4.5×3.5×0.5 |
|  | H12 | 61 | female | adenocarcinoma | 2 | IIIC | T4bN2bM0 | - |
|  | I1 | 61 | female | adenocarcinoma | 2 | IVB | T4aN0M1b | - |
|  | I2 | 51 | male | villoglandular adenocarcinoma | 2 | IVB | T4aN1bM1c | - |
|  | I3 | 70 | male | adenocarcinoma | 2 | IVA | T3N0M1a | 5.0×3.5×1.0 |
|  | I4 | 50 | male | tubular villoglandular adenocarcinoma | 2 | IVA | T3N2aM1a | - |
| D062Co01 | A1 | 68 | male | adenocarcinoma | 2 | IIIB | T3N1aM0 | 3×4×1 |
|  | A2 | 41 | female | mucinous adenocarcinoma | 2 | IIIB | T3N2aM0 | - |
|  | A3 | 57 | male | mucinous adenocarcinoma | 2 | IIIC | T4aN2bM0 | 4×3.5×0.8 |
|  | A4 | 50 | male | adenocarcinoma | 2 | IIIC | T4bN2bM0 | 4×3.5×1.5 |
|  | A5 | 39 | male | mucinous adenocarcinoma | 2 | IIIB | T3N1aM0 | 4.5×4×2.5 |
|  | A6 | 60 | male | adenocarcinoma | 2 | IIIB | T3N1bM0 | 4×3.5×2.5 |
|  | A7 | 58 | male | mucinous adenocarcinoma | 2 | IIIB | T3N1bM0 | 4×3×2 |
|  | A8 | 53 | male | mucinous adenocarcinoma | 2 | I | T2N0M0 | 7×4×2 |
|  | A9 | 63 | male | adenocarcinoma | 2 | IIA | T3N0M0 | 10×9×2.5 |
|  | B1 | 66 | male | mucinous adenocarcinoma | 2 | IIIC | T4aN2aM0 | 5×4×1 |
| D062Co01 | B2 | 60 | male | adenocarcinoma | 2 | IIIB | T3N2aM0 | 7×4×1.4 |
|  | B3 | 54 | male | adenocarcinoma | 2 | I | T2N0M0 | 8×6×6 |
|  | B4 | 54 | female | adenocarcinoma | 2 | IIIC | T4aN2bM0 | 9×4×1 |
|  | B5 | 51 | female | adenocarcinoma | 2 | IIB | T4aN0M0 | 7×6×1.5 |
|  | B6 | 51 | female | adenocarcinoma | 2 | IIA | T3N0M0 | 10×6×1.5 |
|  | B7 | 50 | male | mucinous adenocarcinoma | 2 | IIIA | T2N1aM0 | 4×2×1.5 |
|  | B8 | 58 | male | adenocarcinoma | 3 | IIIB | T3N1bM0 | - |
|  | B9 | 76 | male | adenocarcinoma | 2 | I | T2N0M0 | 7.5×4×3 |
|  | C1 | 53 | male | adenocarcinoma | 2-3 | IIIB | T4aN1bM0 | 4×3×1 |
|  | C2 | 69 | male | mucinous adenocarcinoma | 2 | IIIB | T3N1bM0 | - |
|  | C3 | 45 | female | mucinous adenocarcinoma | 2 | IIIB | T4aN1bM0 | 4×4×1.5 |
|  | C4 | 68 | female | adenocarcinoma | 2 | IIA | T3N0M0 | 6×4×1 |
|  | C5 | 69 | male | adenocarcinoma | 2 | IIIC | T4aN2bM0 | 8.5×6×1.3 |
|  | C6 | 72 | male | mucinous adenocarcinoma | 2 | IIIB | T3N1bM0 | - |
|  | C7 | 69 | male | adenocarcinoma | 2 | IIB | T4aN0M0 | 9×5.5×1.5 |
|  | C8 | 51 | male | adenocarcinoma | 2 | IVA | T2N2aM1a | 4×4×1 |
|  | C9 | 65 | male | mucinous adenocarcinoma | 3 | IIIB | T3N1bM0 | - |
|  | D1 | 51 | female | mucinous adenocarcinoma | 3 | IIA | T3N0M0 | 4×3×1 |
|  | D2 | 67 | female | adenocarcinoma | 2 | IIA | T3N0M0 | 7×6×2 |
|  | D3 | 40 | male | mucinous adenocarcinoma | 2 | I | T2N0M0 | 4×2.5×1.5 |
|  | D4 | 74 | male | mucinous adenocarcinoma | 3 | IVC | T4aN0M1c | - |
|  | D5 | 44 | female | poorly cohesive adenocarcinoma | 3 | IIB | T4aN0M0 | 7×4×2 |
|  | D6 | 54 | male | mucinous adenocarcinoma | 3 | IIIC | T4aN2bM0 | 7×5×2c |
|  | D7 | 72 | female | adenocarcinoma | 3 | IIA | T3N0M0 | - |
|  | D8 | 55 | female | adenocarcinoma | 3 | IIA | T3N0M0 | - |
|  | D9 | 42 | female | mucinous adenocarcinoma | 3 | IIIB | T3N1aM0 | - |
|  | E1 | 69 | male | adenocarcinoma | 2 | IIA | T3N0M0 | 7×3.5×2 |
|  | E2 | 47 | female | adenocarcinoma | 3 | IIA | T3N0M0 | 5×5×2 |
|  | E3 | 79 | male | adenocarcinoma | 2 | IIB | T4aN0M0 | 6×5.5×2 |
|  | E4 | 50 | male | adenocarcinoma | 2 | IIB | T4aN0M0 | 4×3.5×3 |
|  | E5 | 46 | female | mucinous adenocarcinoma | 3 | IIIC | T3N2bM0 | 5.5×4×1.5 |
|  | E6 | 53 | female | mucinous adenocarcinoma | 2 | IIA | T3N0M0 | 5×4×2 |
|  | E7 | 66 | female | adenocarcinoma | 2 | IIIB | T3N1aM0 | 5×5 |
|  | E9 | 55 | female | mucinous adenocarcinoma | 2 | IIIB | T4aN1bM0 | 4.5×4×1 |
|  | F1 | 51 | female | mucinous adenocarcinoma | 2-3 | IIB | T4aN0M0 | 13×4.5×2.5 |
|  | F2 | 84 | male | mucinous adenocarcinoma | 3 | IIB | T4aN0M0 | 5.5×5×2 |
|  | F3 | 72 | female | mucinous adenocarcinoma | 2 | IIA | T3N0M0 | 6.5×6×3 |
| D062Co01 | F4 | 66 | female | adenocarcinoma | 2 | IIIB | T3N1aM0 | 4×4 |
|  | F5 | 50 | male | poorly cohesive adenocarcinoma | 3 | IIA | T3N0M0 | 4×3 |
|  | F6 | 52 | female | adenocarcinoma | 2-3 | IIIB | T4aN1aM0 | 5.5×5.5×2 |
|  | F7 | 66 | male | mucinous adenocarcinoma | 2-3 | I | T2N0M0 | 8×7×2 |
|  | F8 | 53 | male | mucinous adenocarcinoma | 3 | IIA | T3N0M0 | 7×8 |
|  | F9 | 74 | female | adenocarcinoma | 2-3 | IIIB | T3N1aM0 | 5×5×1 |
|  | G2 | 55 | female | adenocarcinoma | 2 | IIIB | T3N2aM0 | 4×3.5×0.5 |
|  | G3 | 53 | male | adenocarcinoma | 2 | IIA | T3N0M0 | 3×3×1 |
|  | G4 | 67 | male | mucinous adenocarcinoma | 3 | IIIB | T3N1bM0 | - |
|  | G5 | 35 | male | normal colon tissue | - | - | - | - |
|  | G6 | 21 | female | normal colon tissue | - | - | - | - |
|  | G7 | 23 | male | normal colon tissue | - | - | - | - |
|  | G8 | 39 | female | normal colon tissue | - | - | - | - |
