# Supplementary figures and images for: Transferrin receptor 1 nuclear translocation facilitates tumor progression via p53-mediated chromatin interactions and genome-wide alterations
Source: Signal Transduct Target Ther. 2025 Jul 8;10:212. doi: 10.1038/s41392-025-02297-6 (PMC12234710; doi:10.1038/s41392-025-02297-6)

**Uncropped images of western blots and gels**

**
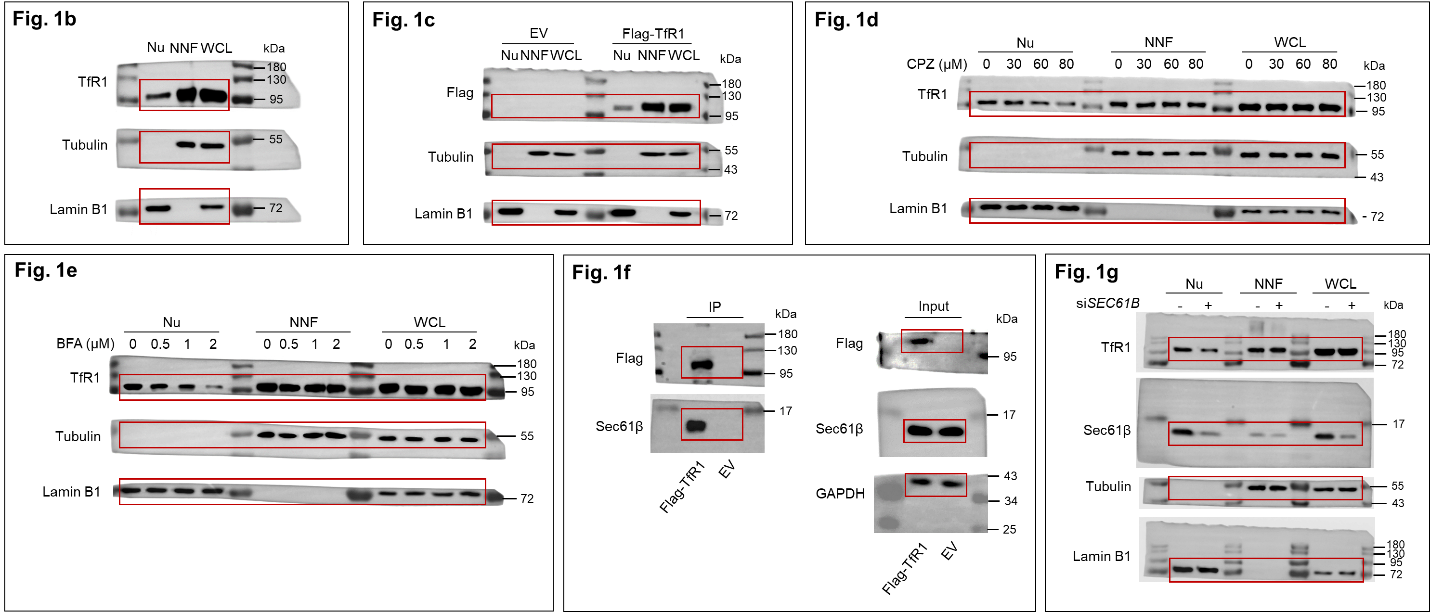
**

**
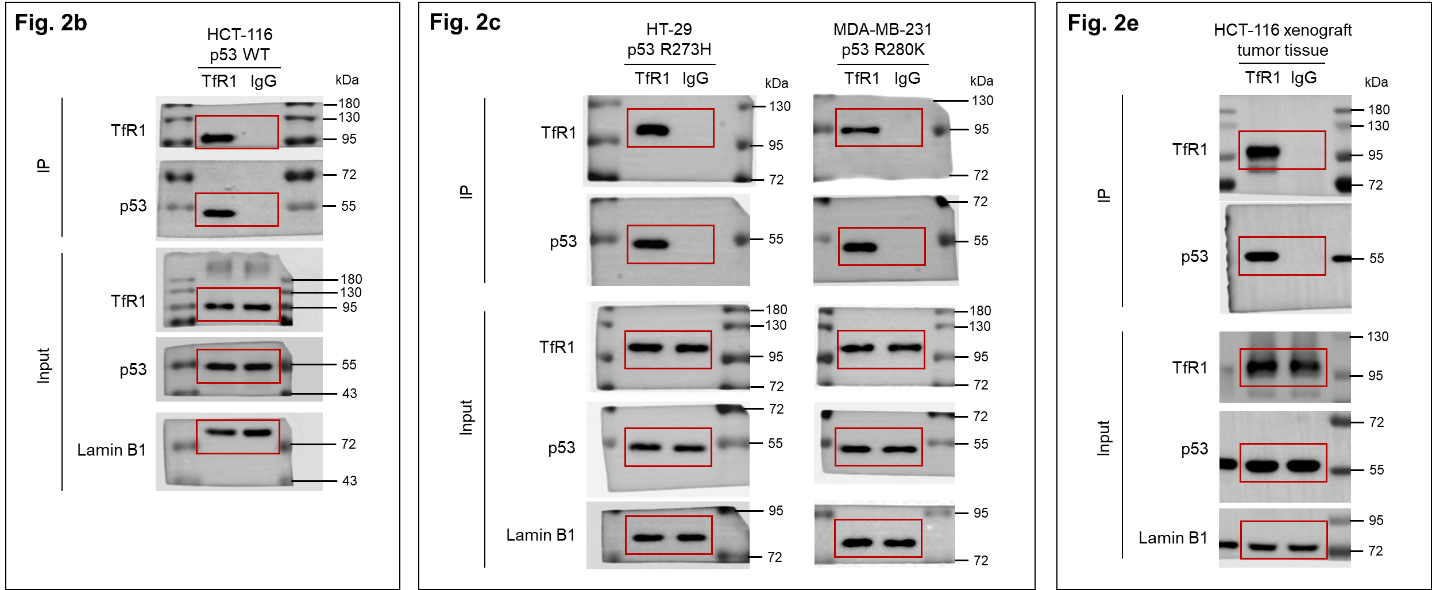
**

**
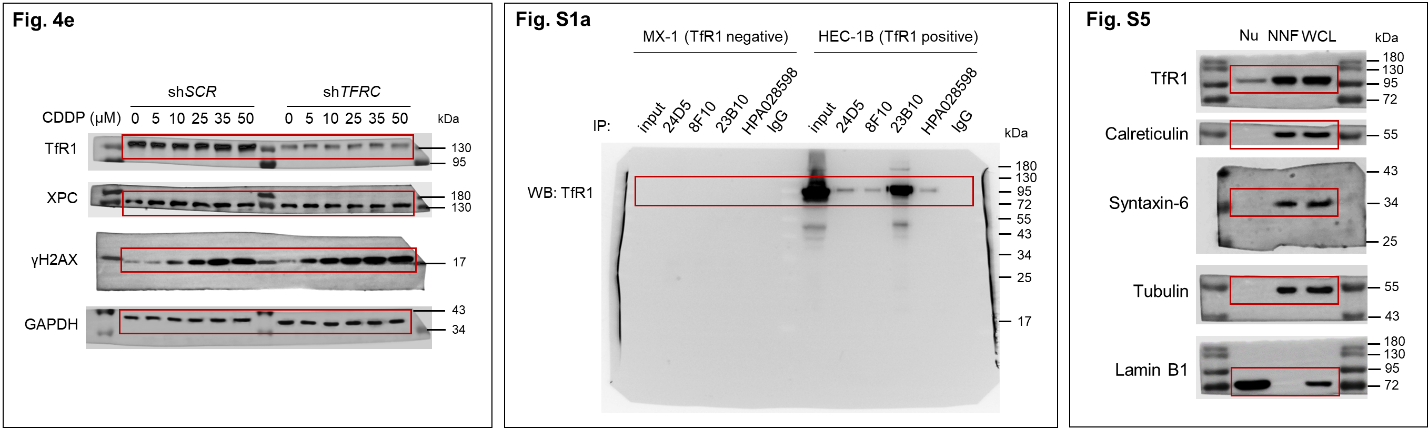
**

**
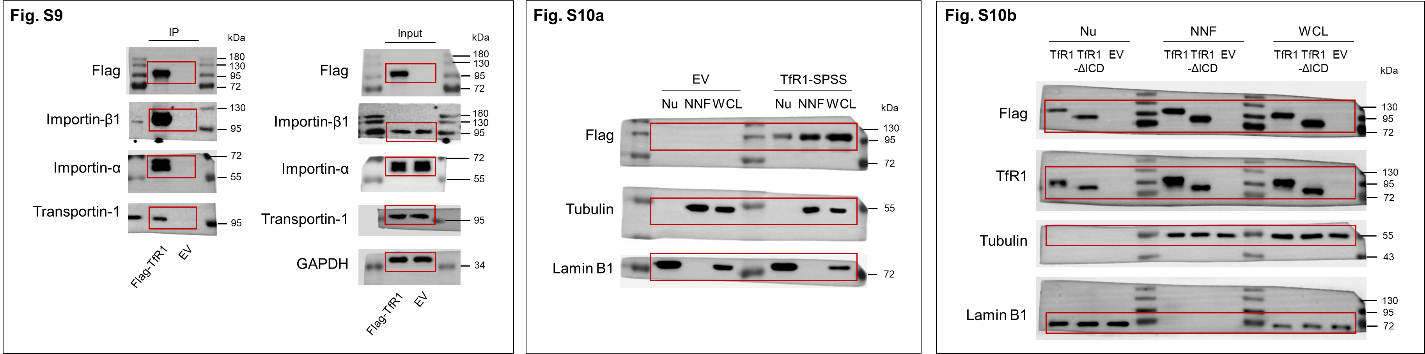
**

**
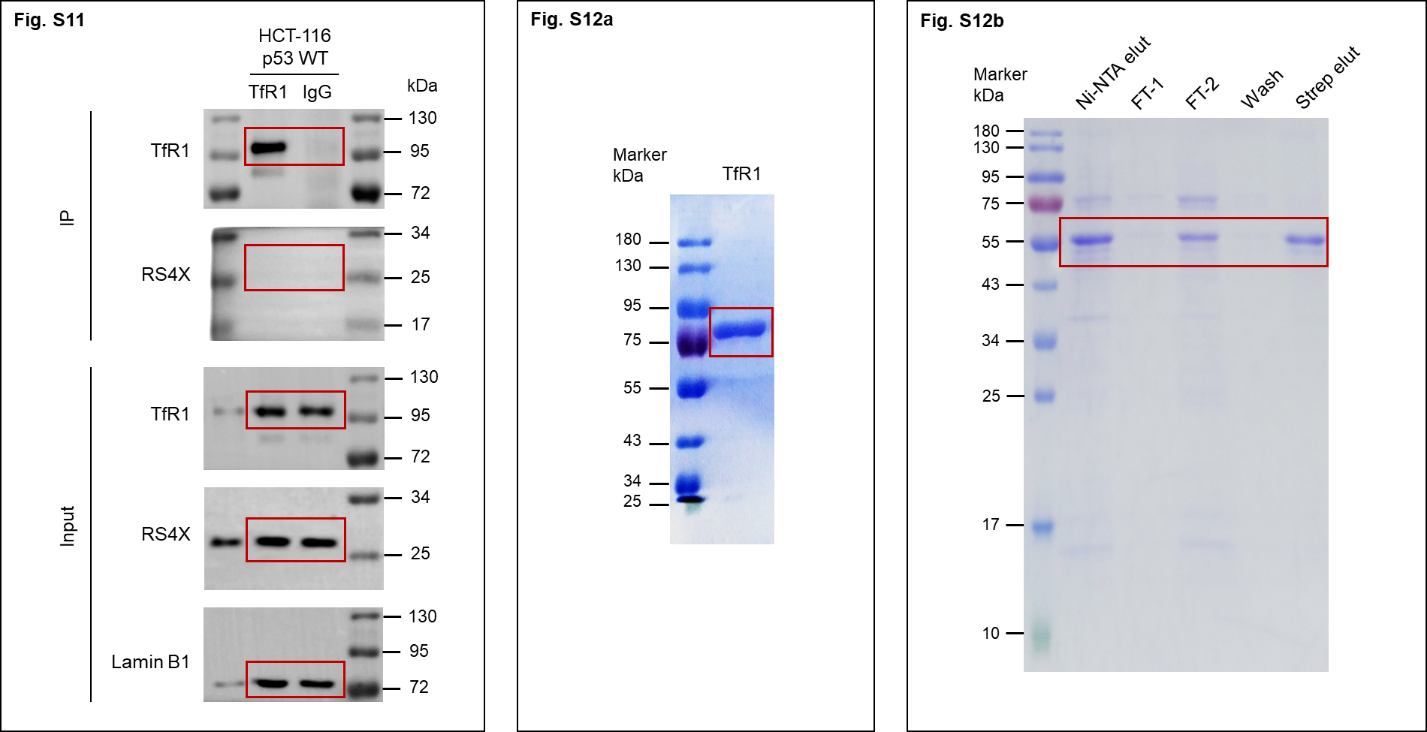
**

**
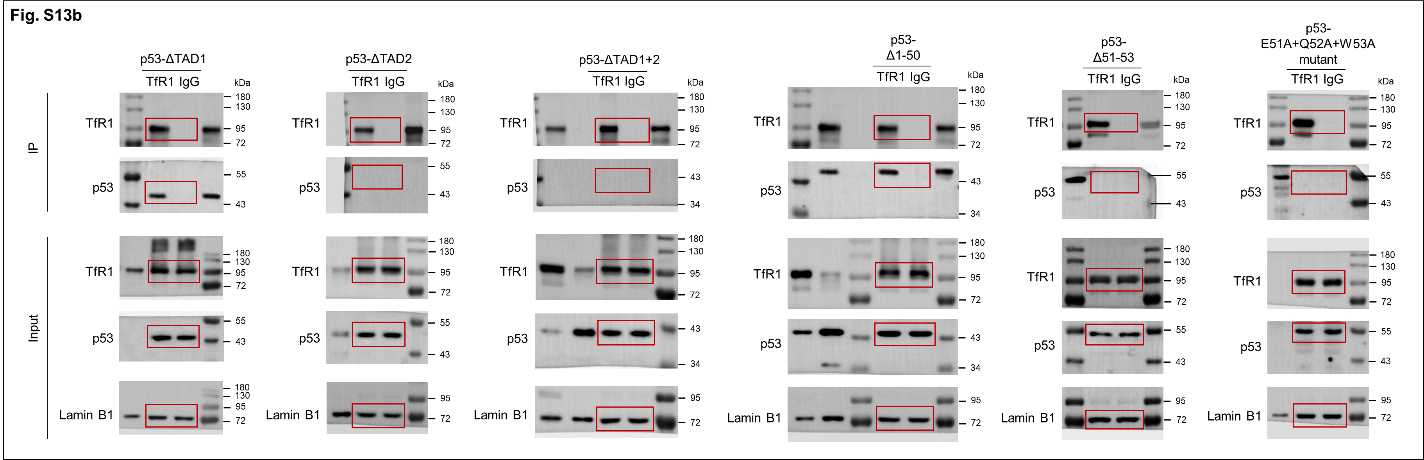
**

**
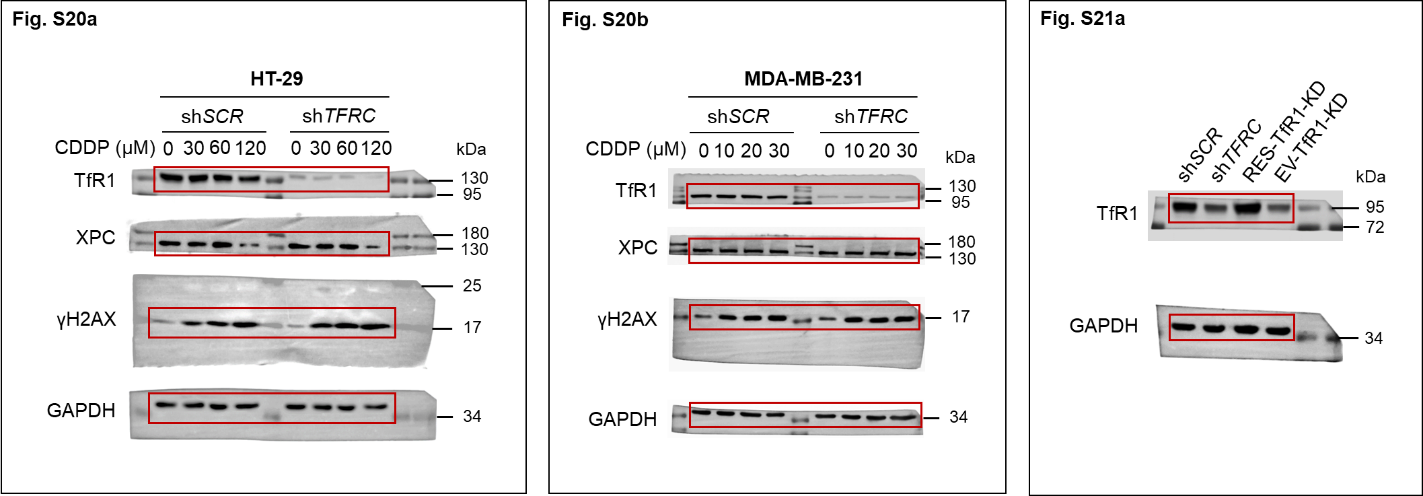
**

**
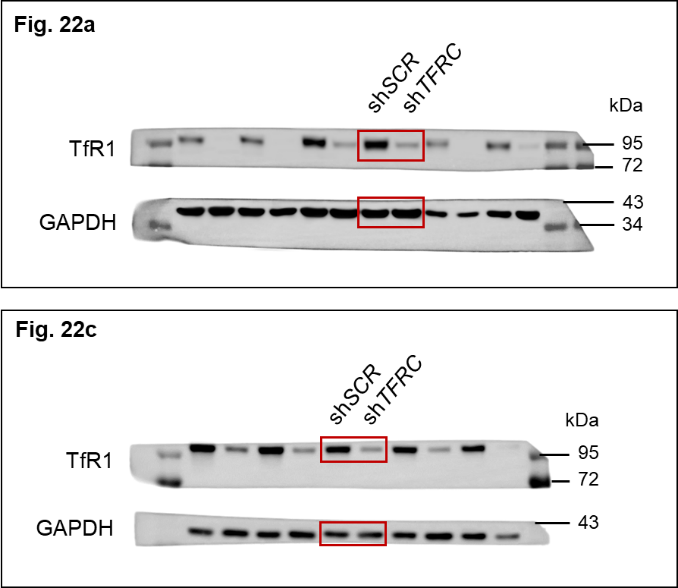
**

Supplement: Supplementary file 2 — Uncropped images of Western Blots and Gels [file 41392_2025_2297_MOESM2_ESM.docx]
